# Neuronal hyperactivity due to loss of inhibitory tone in *APOE4* mice lacking Alzheimer’s disease-like pathology

**DOI:** 10.1038/s41467-017-01444-0

**Published:** 2017-11-13

**Authors:** Tal Nuriel, Sergio L. Angulo, Usman Khan, Archana Ashok, Qiuying Chen, Helen Y. Figueroa, Sheina Emrani, Li Liu, Mathieu Herman, Geoffrey Barrett, Valerie Savage, Luna Buitrago, Efrain Cepeda-Prado, Christine Fung, Eliana Goldberg, Steven S. Gross, S. Abid Hussaini, Herman Moreno, Scott A. Small, Karen E. Duff

**Affiliations:** 1 Taub Institute for Research on Alzheimer’s Disease and the Aging Brain, Columbia University Medical Center, 630 West 168th Street, New York, NY 10032 USA; 2 Department of Pathology and Cell Biology, Columbia University Medical Center, 630 West 168th Street, New York, NY 10032 USA; 3 Department of Neurology and Physiology/Pharmacology, SUNY Downstate Medical Center, The Robert F. Furchgott Center for Neural and Behavioral Science, Brooklyn, NY 11203 USA; 4 Department of Neurology, Columbia University Medical Center, 630 West 168th Street, New York, NY 10032 USA; 5 Department of Pharmacology, Weill Cornell Medical College, 1300 York Avenue, New York, NY 10065 USA; 6 Department of Psychiatry, Division of Integrative Neuroscience, New York State Psychiatric Institute, New York, NY 10032 USA

## Abstract

The ε4 allele of apolipoprotein E (*APOE*) is the dominant genetic risk factor for late-onset Alzheimer’s disease (AD). However, the reason *APOE4* is associated with increased AD risk remains a source of debate. Neuronal hyperactivity is an early phenotype in both AD mouse models and in human AD, which may play a direct role in the pathogenesis of the disease. Here, we have identified an *APOE4*-associated hyperactivity phenotype in the brains of aged *APOE* mice using four complimentary techniques—fMRI, in vitro electrophysiology, in vivo electrophysiology, and metabolomics—with the most prominent hyperactivity occurring in the entorhinal cortex. Further analysis revealed that this neuronal hyperactivity is driven by decreased background inhibition caused by reduced responsiveness of excitatory neurons to GABAergic inhibitory inputs. Given the observations of neuronal hyperactivity in prodromal AD, we propose that this *APOE4*-driven hyperactivity may be a causative factor driving increased risk of AD among *APOE4* carriers.

## Abstract (web summary)

The APOE4 allele is the leading risk factor for late-onset Alzheimer’s disease, but how it might contribute to the disease is not clear. Here the authors show that a mouse expressing the human APOE4 allele displays hyperactivity in the entorhinal cortex due to a decreased inhibitory tone, which may in part explain accelerated Alzheimer’s pathology in APOE4 carriers.

## Introduction

Carriers of the ε4 allele of apolipoprotein E (*APOE*) are at significantly greater risk of developing late-onset Alzheimer’s disease (AD)^1^. In normal physiology, the apoE protein plays a vital role in the transport of cholesterol and other lipids through the bloodstream, as well as within the brain^2–4^. However, the reason why *APOE4* carriers have a higher incidence of AD compared to non-carriers is not understood. While much research has focused on the ability of apoE4 to increase the aggregation and decrease the clearance of Aβ^5–11^, possession of an *APOE4* allele also affects a wide array of additional processes in the brain (see reviews by Huang^12^ and Wolf et al.^13^), and it is unclear if these other functions may play a role in the pathogenesis of AD among *APOE4* carriers.

Recent studies have shown that numerous transgenic mouse models of AD manifest early and pronounced neuronal hyperactivity in AD-vulnerable brain regions such as the hippocampus^14–17^. In addition, functional magnetic resonance imaging (fMRI) studies have shown that humans with mild cognitive impairment (MCI)^18–22^, as well as presymptomatic carriers of familial AD (FAD) mutations^23, 24^, display increased activity in these same regions. Given the link between increased brain activity and accelerated AD pathology^25–30^, this has led to speculation that the observed increase in brain activity early in the pathogenic process may be a driving factor in the development of AD.

With this in mind, it is important to understand if neuronal hyperactivity may be relevant to the pathobiology of *APOE4* carriers. To that end, several neuroimaging studies have examined the effects of *APOE4* on human brain metabolism. In particular, several studies utilizing fMRI to measure task-based brain activity in *APOE4* vs. non-carriers have reported increased blood oxygen level-dependent responses in *APOE4* carriers^31, 32^. However, numerous other task-based fMRI studies have found contradictory findings, regardless of age or family history of AD (see review by Trachtenberg et al.^33^). Interestingly, two other studies that measured resting state brain activity found increased cerebral blood flow in the hippocampal region of cognitively normal, middle aged and elderly *APOE4* carriers^34, 35^.

Since *APOE4* is known to accelerate AD pathology, it is not clear if these neuroimaging findings are a direct result of *APOE4* expression, or if they reflect an interaction with incipient disease, especially as it is now known that the pathophysiological process of AD starts decades before the onset of symptoms^36^. Therefore, to distinguish the *APOE* variant-specific effects on brain metabolism from those caused by AD pathology, we used a well-established mouse model that expresses the human *APOE3* or *APOE4* gene in place of the mouse *Apoe* gene, but which does not develop the plaques and tangles present in AD brain^37, 38^. In order to analyze the neuronal activation state in these mice, we utilized four complementary techniques—fMRI, in vitro electrophysiology, in vivo electrophysiology, and metabolomics. The results from each of these analyses reveal an increase in brain metabolism/activity in the hippocampal formation of aged *APOE4* mice, most notably in the entorhinal cortex (EC). We also report a decrease in the inhibitory tone of excitatory neurons in the EC of aged *APOE4* mice, which is likely to mediate the observed hyperactivity in these mice. We hypothesize that this *APOE4*-associated circuit imbalance may contribute to the pathogenesis of AD among *APOE4* carriers.

## Results

### Hypermetabolism in the hippocampal formation of *APOE4* mice

To test the effects of *APOE4* on brain metabolism in the absence of AD pathology, we studied young (mean age = 8 months; 6 *APOE3*, and 8 *APOE4* males) and old (mean age = 20 months; 7 *APOE3*, and 8 *APOE4* males) *APOE*-targeted replacement mice, which express the human *APOE* gene in place of the endogenous mouse *Apoe* gene^37, 38^. We utilized a high-resolution variant of fMRI^39, 40^ that relies on basal cerebral blood volume (CBV), an established indicator of basal brain metabolism^41–43^. CBV possesses excellent spatial resolution, which is useful for visualizing specific regions of the brain, including the hippocampal formation (EC, dentate gyrus (DG), CA3 and CA1 subfields, and the subiculum), an early and important site of AD pathology^44^.

**Fig. 1:**
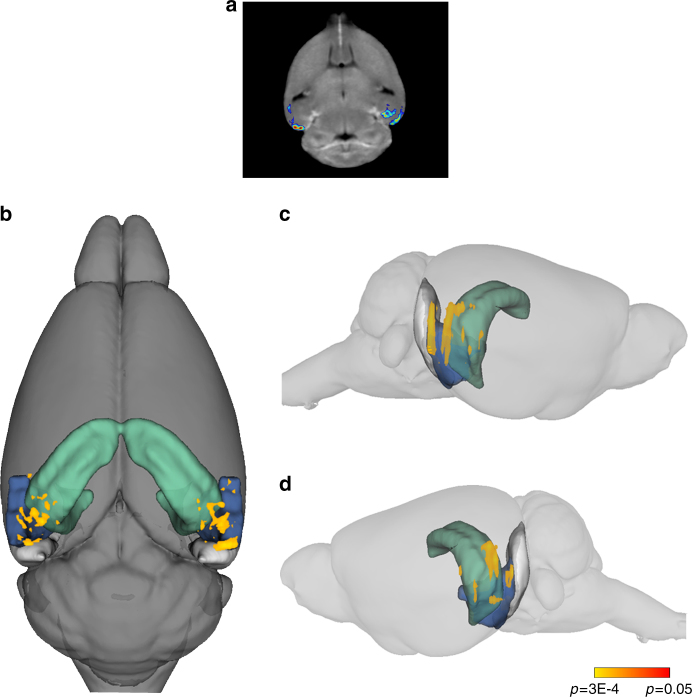
*APOE4* is associated with entorhinal cortex hypermetabolism in aged mice. fMRI analysis was performed on aged *APOE* mice (mean age = 20 months; 7 *APOE3* and 8 *APOE4* males), and a voxel-based analysis was performed to generate CBV maps of the whole brain of each mouse, followed by co-registration of each image, ROI selection of the hippocampal formation, and statistical comparison between genotypes by a Student’s *t* test, followed by multiple test correction. **a** Voxel-based analyses revealed that aged *APOE4* mice had increased CBV in the hippocampal formation compared with *APOE3* mice, primarily centered around the EC. **b** 3D superior view of CBV increase detected in aged *APOE4* vs. *APOE3* mice, as indicated using a heat scale by *p* value. **c** Right sagittal view of the same analysis. **d** Left sagittal view of the same analysis. For each panel, the colored regions depict the hippocampus and subiculum in green, the lateral entorhinal cortex (LEC) in blue, and the medial entorhinal cortex (MEC) in gray

Whole-brain CBV maps were generated with the steady-state gadolinium-enhanced fMRI technique, as previously described^40^, followed by segmentation of the hippocampal formation region of interest (ROI) using operational criteria described previously^45^. An analysis of variance (ANOVA) revealed no significant differences for the younger mice, but a significant difference was detected in the hippocampal formation of the older mice. Compared to *APOE3* mice, aged *APOE4* mice were found to have higher CBV values in both the left and right hippocampal formations (Fig. 1), with the majority of the hypermetabolism centered around the lateral region of the EC, with additional hypermetabolism detected in the medial EC, the subiculum, and the CA1 region of the hippocampus (Fig. 1b, c).

### EC hyperexcitability in awake freely moving *APOE4* mice

**Fig. 2:**
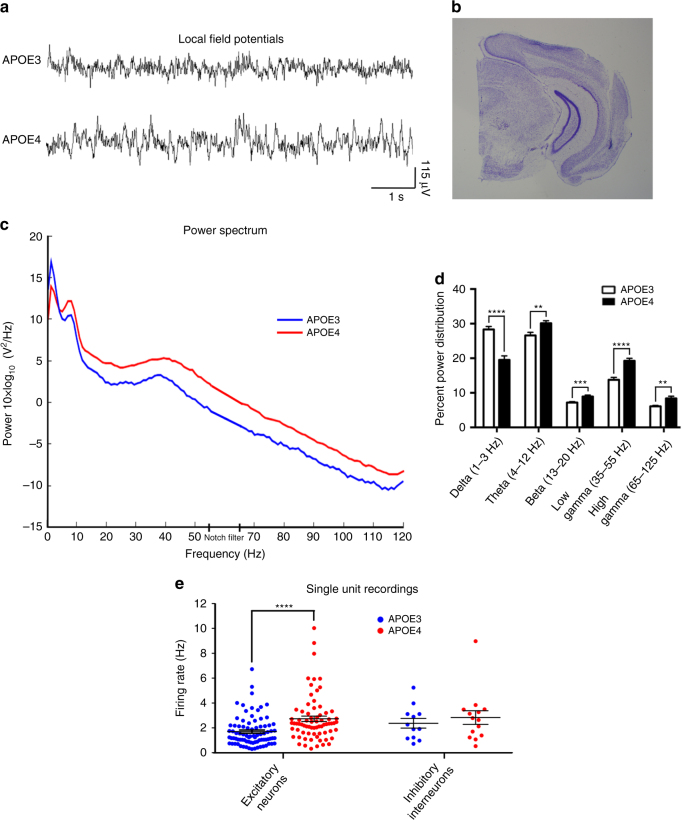
*APOE4* is associated with increased neuronal activity in awake freely moving mice. In vivo electrophysiology was performed on aged *APOE* mice (mean age = 18 months; 5 *APOE3* and 4 *APOE4* males) using a 16-channel microdrive placed directly into the LEC. **a** Representative local field potentials (LFPs) in awake, freely moving (5–20 cm/s) *APOE3* and *APOE4* mice, recorded at a depth of 3.3 mm from the brain surface. **b** A Nissl-stained brain section from a mouse used in this experiment, showing a track mark made by the implanted tetrode, which was descended to a depth of 3.3 mm from the brain surface when the mouse was killed. **c** Power spectra of LFPs recorded at a depth of 3.1–3.3 mm from the brain surface in freely moving (5–20 cm/s) *APOE3* and *APOE4* mice. (Frequencies between 55–65 Hz were notch-filtered to remove electrical noise and are extrapolated in the graph using straight lines.) **d** Percent power distributions comparing LFPs recorded at a depth of 3.1–3.3 mm from the brain surface in freely moving (5–20 cm/s) *APOE3* and *APOE4* mice (mean values and SEM). A Student’s *t* test with Welch’s correction was used for statistical comparison between genotypes (delta, *p* < 0.0001; theta, *p* = 0.0028; beta, *p* = 0.0008; low gamma, *p* < 0.0001; high gamma, *p* = 0.0015). **e** Single unit recordings detected at a depth of 3.1–3.3 mm from the brain surface in *APOE3* and *APOE4* mice, separated into putative excitatory neurons and interneurons based on peak size (black lines represent mean values and SEM). An increased firing rate of excitatory neurons (*p* < 0.0001) was observed using a Student’s *t* test with Welch’s correction. (**p < 0.01, ***p < 0.001, ****p < 0.0001)

In order to confirm and expand upon the fMRI findings, in vivo electrophysiology was performed in awake, freely moving *APOE* mice using 16-channel microdrives, as previously described^46^ (Fig. 2). Briefly, tetrodes were inserted into the lateral region of the EC of aged *APOE* mice (mean age = 18 months; 5 *APOE3* and 4 *APOE4* males) using previously established coordinates (A/P: 3.4, L/M: 4.3, D/V: 2.5–3.5)^47^ that were independently verified in our laboratory. Recordings were performed twice daily for 10–20 min, and the depth of the tetrode was increased by 100 µm every 2–3 days. To determine the local field potential (LFP) and single unit activity in the EC, recordings from depths of 3.1–3.3 mm were pooled and quantified. For LFP measurements, a speed filter of 5–20 cm/s was applied. As shown in Fig. 2c, d, we observed *APOE4*-specific increases in theta, beta, and gamma oscillations of awake, freely moving mice, indicative of an increase in neuronal activity in this region.

In order to identify the source of these observed increases in theta, beta, and gamma oscillations, we analyzed single unit recordings from this same region in *APOE3* and *APOE4* mice. Putative excitatory neurons and inhibitory interneurons were distinguished based on spike width, as previously described^48^. As shown in Fig. 2e, we observed an increase in the firing rates of excitatory neurons in the EC of *APOE4* mice (2.73 ± 0.22 Hz) vs. *APOE3* mice (1.70 ± 0.13 Hz). This was based on the firing rates of 155 total excitatory neurons (83 from *APOE3* and 72 from *APOE4*) detected at a depth of 3.1–3.3 mm. We did not observe a significant difference in the firing rates of inhibitory interneurons in *APOE4* vs. *APOE3* mice. However, we were only able to detect 26 total inhibitory interneurons (12 from *APOE3* and 14 from *APOE4*) at the target depths, which is likely insufficient to accurately assess the activity of inhibitory interneurons in this region.

### Increased energy-related metabolites in *APOE4* EC

To investigate the neuromolecular basis of the observed hyperactivity in the EC, we performed untargeted and targeted metabolite profiling on brain tissues from aged *APOE* mice (14–15 months old; 8 *APOE3/3*, 9 *APOE3/4*, and 7 *APOE4/4* males) using a previously described mass spectrometry (MS) platform^49^. The EC region was compared to the primary visual cortex (PVC), which is relatively resistant to AD pathology^44, 50, 51^. Small-molecule metabolites (50–1000 Da) from the EC and the PVC were extracted and analyzed by MS, and bioinformatics was performed to compare the levels of both untargeted (Supplementary Tables 1 and 2) and targeted (Tables 1 and 2) metabolites. An initial analysis of untargeted metabolites showed that these metabolites clustered independently of each other based on brain region and *APOE* genotype (Supplementary Fig. 2b, c). Differential expression analysis (false-positive discovery rate (FDR) <0.05) between the *APOE3* and *APOE4* genotypes revealed robust differences in a wide range of untargeted metabolites (Supplementary Fig. 2d).

**Table 1:** Differentially expressed targeted metabolites from the EC of *APOE4* vs. *APOE3* mice

Metabolite	Regulation in E4/4	Fold change	P-value	FDR	Detection mode	CAS number
*Fatty acids*						
Gamma-linolenic acid	Down	1.31	1.19E−03	0.093	Positive	506-26-3
Myristic acid	Down	3.92	0.004	0.118	Positive	544-63-8
Docosahexaenoic acid	Up	1.36	0.011	0.154	Positive	6217-54-5
Stearic acid	Down	1.69	0.011	0.154	Positive	57-11-4
12-Hydroxydodecanoic acid	Down	1.36	0.021	0.213	Positive	505-95-3
Arachidic acid	Down	1.40	0.037	0.230	Negative	506-30-9
Palmitic acid	Down	1.32	0.049	0.243	Negative	57-10-3
*Oligosaccharides*						
Trisaccharide	Up	1.99	0.001	0.049	Negative	512-69-6
Tetrasaccharide	Up	2.24	0.015	0.169	Negative	10094-58-3
Disaccharide	Up	2.20	0.015	0.169	Negative	63-42-3
*Vitamin and vitamin derivatives*						
Phylloquinone	Up	1.47	0.003	0.102	Positive	84-80-0
Tocopherol	Up	2.74	0.005	0.123	Negative	59-02-9
Dehydroascorbic acid	Up	1.76	0.008	0.134	Positive	490-83-5
*Energy-related metabolites*						
Inosine 5′-monophosphate (IMP)	Up	2.26	0.003	0.0720	Negative	131-99-7
d-Fructose 6-phosphate	Up	1.10	0.011	0.169	Negative	643-13-0
Succinoadenosine	Up	1.47	0.011	0.169	Negative	4542-23-8
Carnitine	Up	1.27	0.021	0.213	Positive	541-15-1
Citric acid/isocitric acid	Up	1.24	0.028	0.230	Negative	77-92-9/1637-73-6
Malic acid	Up	1.16	0.037	0.230	Negative	97-67-6
ATP	Up	1.24	0.049	0.243	Negative	56-65-5
*Cholesterol metabolites*						
Lanosterol	Up	2.27	0.015	0.169	Negative	79-63-0
Cholesteryl acetate	Up	2.29	0.015	0.169	Negative	604-35-3
*Amino acids*						
Leucine	Up	1.26	0.015	0.169	Negative	61-90-5
Proline	Up	1.12	0.037	0.230	Negative	147-85-3
Glycine	Up	1.10	0.037	0.230	Negative	56-40-6
*Tryptophan metabolites*						
Quinaldic acid	Up	1.68	0.001	0.049	Negative	93-10-7
Kynurenine	Up	2.49	0.015	0.169	Negative	2922-83-0
Kynurenic acid	Up	1.32	0.049	0.243	Negative	492-27-3
*Cysteine and methionine metabolites*						
S-Adenosylhomocysteine	Up	1.15	0.021	0.213	Positive	979-92-0
*Histidine metabolism*						
Carnosine	Up	1.31	0.037	0.230	Negative	305-84-0
*Arginine and proline metabolites*						
4-Oxoproline	Up	1.30	0.003	0.102	Positive	4347-18-6
*Tyrosine metabolites*						
Vanylglycol (MHPG)	Up	1.64	0.011	0.169	Negative	67423-45-4
Tyramine	Up	1.06	0.028	0.216	Positive	51-67-2
Thymidine	Down	1.49	0.028	0.216	Positive	50-89-5
Uracil	Down	1.38	0.049	0.243	Negative	66-22-8
*Purine metabolism*						
GMP	Up	1.14	0.028	0.230	Negative	85-32-5
*Miscellaneous*						
Hydroxybutyric acid	Up	1.35	0.002	0.055	Negative	5094-24-6
Methylglutarylcarnitine	Up	2.42	0.005	0.134	Positive	102673-95-0
Trimethylamine N-oxide	Up	2.65	0.021	0.213	Positive	1184-78-7
2-Hydroxypyridine	Up	1.36	0.037	0.275	Positive	142-08-5
*N*-Acetylneuraminic acid	Up	1.23	0.037	0.230	Negative	131-48-6

**Table 2:** Differentially expressed targeted metabolites from the PVC of *APOE4* vs. *APOE3* mice

Metabolite	Regulation in E4/4	Fold change	P-value	FDR	Detection mode	CAS number
*Fatty acids*						
Gamma-linolenic acid	Down	1.19	0.003	0.068	Positive	506-26-3
Palmitic acid	Down	1.32	0.021	0.212	Negative	57-10-3
10-Hydroxydecanoate	Up	2.22	0.015	0.195	Positive	1679-53-4
Docosahexaenoic acid	Down	2.03	0.021	0.212	Negative	6217-54-5
Arachidonic acid	Down	1.45	0.021	0.212	Negative	506-32-1
*Oligosaccharides*						
Trisaccharide	Up	1.82	0.015	0.212	Negative	
Tetrasaccharide	Up	2.70	0.021	0.212	Negative	
*Vitamin and vitamin derivatives*						
Dehydroascorbic acid	Up	2.03	0.001	0.062	Positive	490-83-5
Phylloquinone	Up	1.90	0.001	0.062	Positive	84-80-0
Ascorbic acid	Up	105.84	0.005	0.192	Negative	50-81-7
Tocopherol	Up	2.28	0.008	0.192	Negative	59-02-9
*Energy-related metabolites*						
Acetylcarnitine	Up	1.40	0.003	0.068	Positive	3040-38-8
Carnitine	Up	1.31	0.011	0.154	Positive	541-15-1
Coenzyme A (CoA)	Up	2.82	0.028	0.282	Positive	85-61-0
Pyruvate	Up	1.43	0.028	0.238	Negative	127-17-3
*Cholesterol metabolites*						
Taurodeoxycholate	Up	1.94	0.003	0.068	Positive	516-50-7
Cholesteryl acetate	Up	2.24	0.008	0.192	Negative	604-35-3
Lanosterol	Up	2.50	0.011	0.192	Negative	79-63-0
*Amino acids*						
Tyrosine	Up	1.25	0.037	0.270	Negative	60-18-4
Serine	Down	1.18	0.049	0.270	Negative	56-45-1
Glutamate	Down	1.08	0.049	0.282	Positive	56-86-0
*Cysteine and methionine metabolites*						
Pterin	Down	3.91	0.002	0.192	Negative	2236-60-4
*Histidine metabolites*						
Urocanic acid	Down	1.34	0.021	0.212	Negative	104-98-3
Carnosine	Up	1.32	0.028	0.282	Positive	305-84-0
*Arginine and proline metabolites*						
Phosphocreatine	Up	1.62	0.037	0.282	Positive	67-07-2
4-Guanidinobutyric acid	Down	1.09	0.049	0.270	Negative	463-00-3
*Threonine metabolites*						
2-Ketobutyric acid	Up	1.43	0.021	0.212	Negative	600-18-0
*Tyrosine metabolites*						
Homovanillic acid	Down	1.30	0.049	0.270	Negative	306-08-1
*Riboflavin metabolites*						
2,4-Dihydroxypteridine (lumazine)	Up	2.07	0.008	0.154	Positive	487-21-8
Flavin adenine dinucleotide (FAD)	Up	1.19	0.021	0.212	Negative	146-14-5
Lumichrome	Down	1.71	0.049	0.282	Positive	1086-80-2
*Pyrimidine metabolites*						
2-Aminoisobutyric acid	Up	1.38	0.011	0.154	Positive	62-57-7
UMP	Up	1.22	0.049	0.270	Negative	58-97-9
CMP	Up	1.29	0.049	0.282	Positive	63-37-6
*Miscellaneous*						
Lipoic acid	Up	3.76	0.005	0.192	Negative	1077-28-7
Thiourea	Down	1.45	0.008	0.192	Negative	62-56-6
Butyrylcarnitine	Up	1.24	0.011	0.154	Positive	25576-40-6
Hydroxybutyric acid	Up	1.49	0.011	0.192	Negative	
Indoxyl sulfate	Down	2.12	0.021	0.212	Negative	2642-37-7
3-Hydroxymethylglutaric acid	Up	1.27	0.028	0.238	Negative	503-49-1
2-Hydroxypyridine	Up	1.45	0.037	0.282	Positive	142-08-5
Maleic acid	Up	1.92	0.037	0.270	Negative	110-16-7
*N*-Acetylneuraminic acid	Up	1.17	0.037	0.282	Positive	131-48-6
*N*-Methylglutamic acid	Down	1.19	0.049	0.270	Negative	35989-16-3
Cytidine diphosphate choline (CDP choline)	Down	1.17	0.049	0.282	Positive	987-78-0
Glutathione	Up	1.49	0.049	0.282	Positive	70-18-8

The targeted analysis, in which metabolites were matched against a proprietary database of 626 biologically relevant metabolites, revealed *APOE4*-specific differential expression (*p* < 0.05) of several important metabolite groups in the EC (Table 1), including fatty acids (gamma-linoleic acid, myristic acid, docosahexaenoic acid, 12-hydroxydodecanoic acid, stearic acid, arachidic acid, and palmitic acid; all downregulated in *APOE4* EC except docosahexaenoic acid), vitamin or vitamin derivatives (phylloquinone, dehydroascorbic acid, and tocopherol; all upregulated in *APOE4* EC), and C_6_H_12_O_6_-subunit containing oligosaccharides (disaccharide, trisaccharide, and tetrasaccharide; all upregulated in *APOE4* EC). The majority of these fatty acids, vitamins and oligosaccharides were similarly regulated in the PVC (Table 2).

**Fig. 3:**
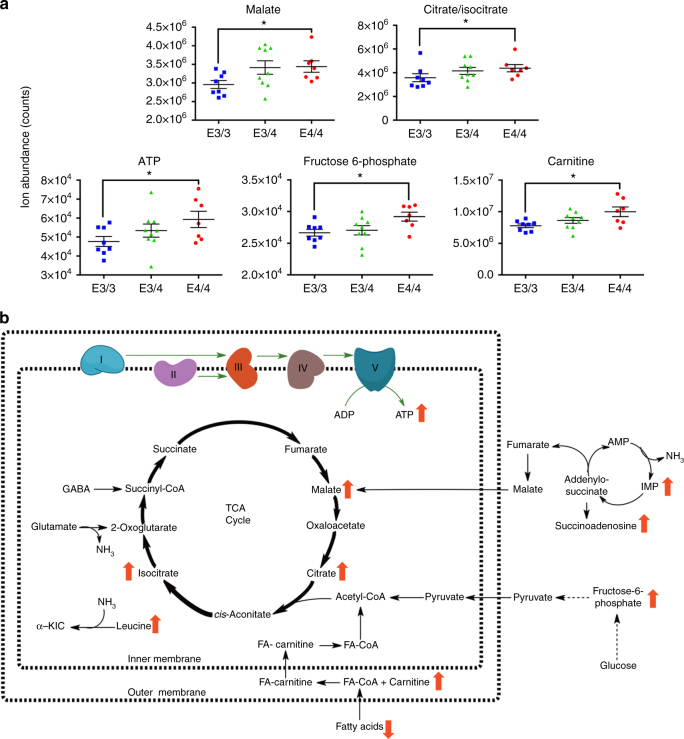
*APOE4* is associated with increased expression of small molecules related to energy metabolism. Targeted metabolite profiling was performed on small-molecule metabolites extracted from the EC and PVC of aged *APOE* mice (14–15 months old; 8 *APOE3/3*, 9 *APOE3/4*, and 7 *APOE4/4* males). **a** Scatter dot plots depicting metabolite abundance in the EC for five energy-related metabolites found to be upregulated (Mann–Whitney; malate, *p* = 0.037; citrate/isocitrate, *p* = 0.028; ATP, *p* = 0.049; fructose-6-phosphate, *p* = 0.011; carnitine, *p* = 0.021) in *APOE4* EC (black lines represent mean values and SEM). **b** Schematic of the energy metabolism-related metabolites found to be dysregulation in *APOE4* EC. (*p ≤ 0.05)

Critical to this study, we also observed an *APOE4*-specific upregulation of a number of mitochondrial metabolites that are involved in cellular energy metabolism (Fig. 3). These include several TCA-cycle metabolites (malate, citrate, and isocitrate), fructose-6-phosphate, which is an important glycolysis intermediate, and carnitine, which is required for the transport of fatty acids into the mitochondrial matrix for energy utilization via beta-oxidation. Importantly, we also observed an *APOE4*-specific upregulation of ATP. In addition, we observed an *APOE4*-specific upregulation of several metabolites that are involved in mitochondria-related ammonia regulation, including leucine, which is used for ammonia shuttling between astrocytes and neurons, and inosine monophosphate and succinoadenosine, which are involved in the ammonia-generating purine nucleotide cycle (see Fig. 3b for an overall schematic). With the exception of carnitine, which was upregulated in both the EC and PVC and may play a role in the decreased levels of various fatty acids observed in these regions, the other energy-related metabolites were only found to be upregulated in the EC. This *APOE4*-specific increase in energy-related metabolites in the EC represents a molecular correlate to the observed hypermetabolism/hyperexcitability revealed by the previously described fMRI and in vivo electrophysiological experiments.

### Hyperexcitability and reduced inhibitory tone in *APOE4* mice

**Fig. 4:**
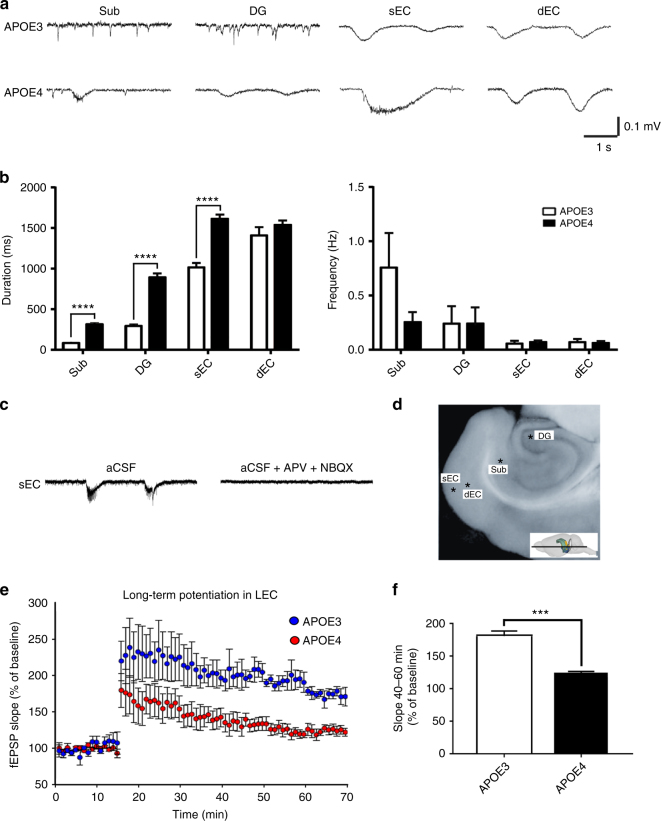
*APOE4* is associated with abnormal excitability and synaptic plasticity in the hippocampal formation. In vitro electrophysiology was performed on horizontal hippocampal brain slices from aged *APOE* mice (mean age = 20 months; 5 *APOE3* and 4 *APOE4* males). **a** Representative traces of spontaneous extracellular field potentials (sEFPs) recorded in the subiculum (Sub), the dentate gyrus (DG), superficial layers (II/III) of the EC (sEC), and deep layers (V/VI) of the EC (dEC) in *APOE3* and *APOE4* mice. **b** Event durations and frequencies comparing *APOE3* and *APOE4* mice in the different regions recorded (mean values and SEM). Event durations in the Sub, DG, and sEC were found to be increased in aged *APOE4* mice (Mann–Whitney; *p* < 0.001). **c** Representative sEFPs in *APOE4* sEC before and after bath application (25 min) of AP-V (30 μM) and NBQX (10 μM). **d** A representative horizontal hippocampal brain slice used in this study, with asterisks denoting the location where each electrode was placed and the insert depicting the location of the horizontal slice in the mouse brain. **e** Cortico-cortical LTP in superficial EC induced by high-frequency stimulation (HFS, 3 trains of 100 pulses at 100 Hz, 10 s interval). Stimulation electrode was placed in lateral EC layer II, and the recording electrode was placed in lateral EC layers III/II. Stimulus intensity was selected to be 50–60% of the maximum amplitude of the fEPSP observed in the input–output curve, and a baseline for 15 mins was obtained before HFS. The slope of the initial component of the fEPSP (10–90%) was normalized to baseline and compared between *APOE3* and *APOE4* mice (repeated measures ANOVA; *F*_(1,6)_ = 15.001, *p* = 0.008). **f** Mean value of potentiation between 40 and 60 min after LTP induction, comparing *APOE3* and *APOE4* (*t*_(6)_ = 3.878, *p* = 0.008). (**p < 0.01)

In order to further study the basis of *APOE4*-associated hyperactivity, we performed in vitro electrophysiology on 400 μM thick horizontal slices consisting of ventral hippocampus, subicular complex, entorhinal, perirhinal, and temporal cortices, prepared as previously described^52^, from aged *APOE* mice (mean age = 20 months; 5 *APOE3* and 4 *APOE4* males). First, we investigated the overall excitability in these slices by placing electrodes in the superficial (II/III) and deep (V/VI) layers of the lateral EC, as well as in the subiculum and DG, from which we recorded spontaneous extracellular field potentials (sEFPs) at 34 °C (Fig. 4). sEFPs occurring at different frequencies were detected (Fig. 4a, b). Compared to the *APOE3* mice, superficial layers of the EC had significantly longer sEFPs in *APOE4* mice (*p* < 0.0001), with no differences in frequency (*p* = 0.58), while no significant differences were observed in the duration of sEFPs in deep layers of the EC between *APOE3* and *APOE4* mice (*p* = 0.11). Additionally, we observed significantly longer sEFPs in both the subiculum (*p* < 0.0001) and the DG (*p* < 0.0001) of *APOE4* mice, with no significant differences in the frequency in the subiculum (*p* = 0.29) or the DG (*p* = 0.85). In every case, we blocked transmission by adding (2*R*)-amino-5-phosphonovaleric acid (APV, 30 μM) and 2,3-dihydroxy-6-nitro-7-sulfamoyl-benzo[f]quinoxaline-2,3-dione (NBQX, 10 μM) to the bath, demonstrating that these are mainly synchronized glutamatergic synaptic events (Fig. 4c). Given the afferent projections from the superficial layers of the EC to the Sub and DG^53, 54^, it is possible that the increased activity observed in the subiculum and DG are a downstream result of the observed EC activation, as has been shown previously in the case of EC to DG activation^53^.

We also studied evoked EC synaptic activity by stimulating EC layer II and recording at EC layer II/III (Fig. 4e, f). After obtaining an input–output relationship and an appropriate baseline, we elicited synaptic long-term potentiation (LTP) by high-frequency stimulation (HFS) in EC layer II, as reported previously^55^. The mean potentiation (late phase) elicited by HFS in *APOE4* mice between 40 and 60 min after HFS was 140 ± 13%, which was significantly lower than in *APOE3* mice (190 ± 15%), suggesting that the electrophysiological differences observed in the EC result in impaired long-term synaptic plasticity in this region.

In order to understand the mechanism responsible for the observed hyperactivity, we performed a series of whole-cell patch-clamp experiments in layer II EC of aged *APOE* mice (mean age = 20 months). Excitatory neurons in this region fall into two distinct electrophysiological and morphological categories: the pyramidal neurons and non-pyramidal neurons such as stellate and fan cells^56, 57^. We concentrated on pyramidal neurons here (*n* = 6 cells per group). Current clamp recordings demonstrated that *APOE3* mice had larger amplitude and wider evoked action potentials (with constant current injection, see Methods section) as compared to APOE4 mice (Supplementary Fig. 3) (amplitude *F* = 88.0, *p* < 0.0001 and width *F* = 19.9, *p* < 0.001). Additionally, we induced focal seizure-like discharges (fSLDs) by direct pressure application of NMDA (1 mM) for 200 ms in the vicinity of the recorded neurons (Supplementary Fig. 4a, b). Simultaneously, calcium-induced fluorescence changes were also monitored upon NMDA application. The results revealed that NMDA-evoked fSLDs were not significantly different between the two groups in the multiple measurements obtained (Supplementary Fig. 4c–e). NMDA-evoked calcium transients, evaluated by changes in fluorescence (Δ*F*) in the soma of EC layer II pyramidal neurons, were also similar between the two groups (Supplementary Fig. 4f). These data suggest that the observed increases in sEFPs in the EC of aged *APOE4* mice are not due to increased excitability of pyramidal cells of this region and may point to a defect in inhibitory signaling as the primary source of this hyperexcitability. Moreover, the reduced amplitude and narrower evoked action potentials observed in the *APOE4* mice may represent an adaptive response to the decreased inhibitory tone of the EC in these mice.

**Fig. 5:**
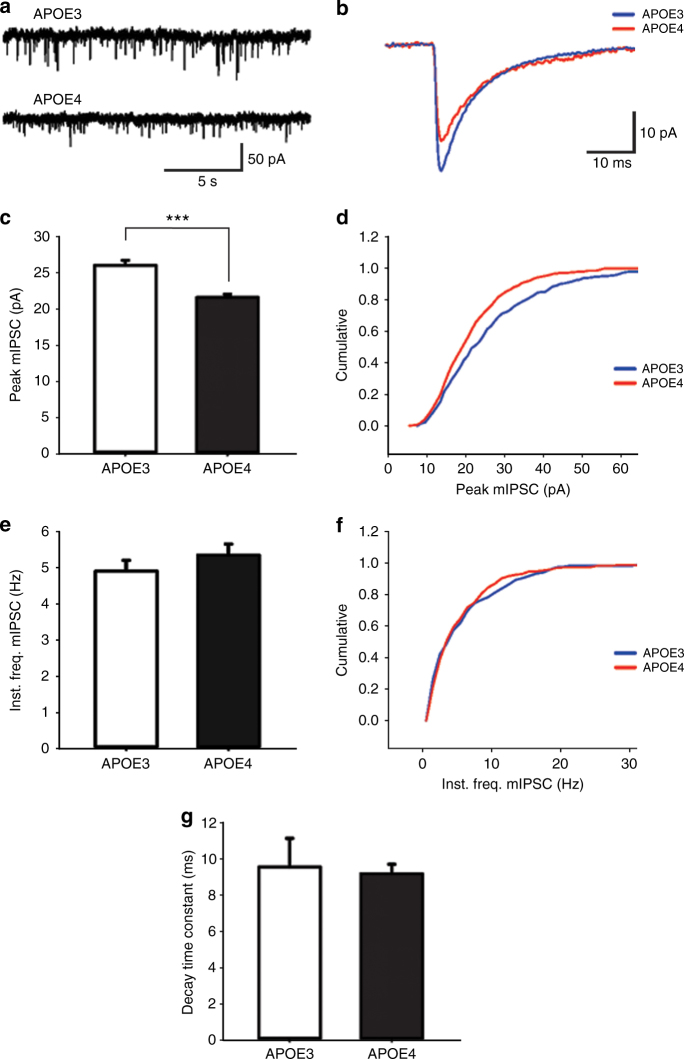
*APOE4* is associated with decreased background synaptic inhibition in layer II entorhinal cortex pyramidal neurons. Miniature inhibitory post-synaptic currents (mIPSCs) were recorded using the patch-clamp whole-cell modality on pyramidal cells (*n* = 7 cells from *APOE3* and *n* = 8 cells from *APOE4*) in the layer II EC of aged *APOE* mice (mean age = 20 months). **a** Representative traces of mIPSCs of layer II EC pyramidal neurons of *APOE3* and *APOE4* mice from recordings in voltage-clamp modality (holding at −65 mV) in the presence of APV (10 µM), NBQX (16 µM), and TTX (1 µM). **b** Representative average mIPSCs from a 3 min recording of an *APOE3* and *APOE4* mouse. **c** Plotted are mean + SEM values of peak amplitude of mIPSCs in the different genotypes as indicated (*APOE3* 26.09 ± 0.73 pA; A*POE4* 21.6 ± 0.48 pA; *p* < 0.001). **d** Cumulative probability distribution of peak amplitude in *APOE3* and *APOE4* mice, showing lower values for *APOE4* in the entire distribution. **e** Shown are mean + SEM values of instantaneous frequencies in the different genotypes (*APOE3* 4.92 ± 0.3 Hz; *APOE4* 5.36 ± 0.31 Hz; *t*(748) = −1.035, *p* = 0.301). **f** Cumulative probability distribution of instantaneous frequencies in *APOE3* and *APOE4* mice, showing no significant differences. **g** Plotted are mean + SEM values of decay time constant in both groups (*APOE3* 9.56 ± 1.61 ms; *APOE4* 9.19 ± 0.53; *p* = 0.819). (***p < 0.001)

In order to investigate this possibility, we recorded miniature inhibitory post-synaptic currents (mIPSCs) in layer II EC pyramidal neurons (*n* = 7 cells from *APOE3* and *n* = 8 cells from *APOE4*) of aged *APOE* mice (mean age = 20 months) (Fig. 5a, b). The peak amplitude of mIPSCs were significantly smaller in the *APOE4* pyramidal neurons as compared to *APOE3* neurons (Fig. 5c). Cumulative probability distributions showed that the entire distribution of mIPSC amplitudes were shifted toward smaller values for *APOE4* mice as compared to *APOE3* (Fig. 5d). Conversely, a comparison of instantaneous frequencies revealed no significant differences between groups (Fig. 5e, f). Individual measurements of decay time constant also showed no significant differences between conditions (Fig. 5g). The lack of effect on frequency and kinetics suggests that there is no significant difference between *APOE4* and *APOE3* in the spatial segregation of inhibitory inputs onto the pyramidal neurons, and in consequence, no difference in electrotonic filtering. The present data suggest that the observed difference in *APOE4* mIPSC amplitude is through a post-synaptic change, either in receptor (GABA-A) number or function, or in overall synaptic number or morphology. We also cannot rule out the possibility that there may be additional presynaptic changes, such as the GABA content in synaptic vesicles. However, this possibility of decreased vesicular GABA content may be difficult to evaluate due to the fact that miniature release is highly variable from synapse to synapse^58^. In summary, mIPSC data indicate that the action potential-independent background synaptic inhibition in layer II of EC is decreased in *APOE4* as compared to *APOE3*, and differences in the properties of such inhibition can explain the hyperexcitability observed in the EC of *APOE4* mice.

### Lack of overt amyloid or tau pathology in *APOE4* mice

The brains of *APOE4* mice have been reported to have slightly elevated levels of Aβ and phosphorylated tau^59^, and older mice (over 18 months) develop some vascular amyloid deposits in the frontal cortex and hippocampus^60^. To examine whether this was apparent in the EC of the aged *APOE4* mice used for the imaging studies, we performed postmortem studies in which brain sections were immunolabeled with an antibody recognizing murine APP/Aβ (4G8) and an antibody against murine phospho-tau (CP13). Results showed no evidence of AD-related histopathology anywhere in the brain (Supplementary Fig. 1a, b).

## Discussion

Neuronal hyperactivity has been demonstrated in AD transgenic mouse models^14–17^, as well as in humans with MCI^18–22^ and in presynaptic carriers of FAD-mutations^23, 24^. In addition, numerous neuroimaging studies have sought to elucidate the effect of *APOE4* on overall brain metabolism, but they have generated contradictory results^33–35^. We hypothesize that some of this contradiction may be due to varying degrees of incipient AD pathology in the brains of the study participants. Therefore, in order to investigate the effects of differential *APOE* gene variants on brain metabolism in a pathology-free environment, we performed CBV-fMRI in young and old transgenic mice expressing human *APOE3* or *APOE4*. This analysis revealed hypermetabolism in the hippocampal formation of aged *APOE4* mice, most notably in the EC. (Fig. 1). Subsequent in vivo electrophysiological analysis revealed an increase in activity in the EC of awake, freely moving aged *APOE4* mice, with LFP recordings showing an *APOE4*-specific increase in theta, beta, and gamma oscillations, and single unit recordings showing an increase in the firing rates of excitatory neurons (Fig. 2). In addition, using an MS-based metabolite profiling platform, we observed an upregulation of numerous small molecules related to energy metabolism in the EC of aged *APOE4* mice, including ATP (Fig. 3). Finally, in vitro electrophysiological analysis revealed increased durations of spontaneous synchronized events in superficial layers of the EC of aged *APOE4* mice, as well as in the subiculum and DG, with a corresponding deficit in LTP in the superficial layers of the EC of these mice (Fig. 4). Additional in vitro electrophysiology revealed a decreased background inhibitory tone on EC pyramidal neurons, likely caused by reduced responsiveness to GABAergic inhibitory inputs.

Each of these four methods—fMRI, in vitro and in vivo electrophysiology, and metabolomics—have strengths and limitations, with each compensating for the limitations of the others. While fMRI is a powerful tool for detecting overall brain metabolism, the direct cause of the observed differences in brain metabolism is not always known. Electrophysiology, on the other hand, is a rigorous method for directly measuring neuronal activity; however, the molecular mechanisms of any detected activity differences can be difficult to discern. And while metabolite profiling can detect specific differences in the levels of hundreds of molecular species, the resulting effects on brain activity and morphology cannot be determined using this method. Cumulatively, however, when the results from these diverse techniques are viewed as a whole, they provide a cohesive and consistent picture of a hypermetabolic/hyperactive state that is present in the EC of aged *APOE4* mice. In addition, the increased firing rate of excitatory neurons revealed by single unit in vivo electrophysiology, combined with the observation of decreased mIPSC amplitudes (but not frequency) recorded via in vitro electrophysiology, reveal a distinct mechanism of hyperactive excitatory neurons with decreased inhibitory tone, likely caused by reduced responsiveness to inhibitory inputs from GABAergic interneurons in the aged *APOE4* EC.

These results corroborate and expand upon previous data that have been reported on these mice. For example, it has been reported that aged *APOE4* mice demonstrate increased excitatory synaptic activity in the amygdala^61^, a region directly connected with the EC. Furthermore, *APOE4* mice have been reported to have an age-associated seizure phenotype^62^, which may be a downstream consequence of the observed hyperactivity in the EC. In addition, a recent report has shown that *APOE4* mice possess an age-dependent reduction in slow gamma oscillations during sharp wave ripples (SWRs)^63^, which are unique high-frequency bursts that occur primarily in the CA1 region of the hippocampus during periods of slow-wave sleep or awake immobility. Interestingly, the timing of SWRs have previously been shown to be affected by activity changes in the EC, either directly or by way of the DG^64^, suggesting that EC hyperactivity may play a role in the observed abnormalities in slow gamma oscillations during SWRs.

Increased brain activity has been shown to accelerate AD pathology. In addition to causing increased production and secretion of Aβ^25–27^, increased neuronal activity may also affect tau pathogenesis, as tau release from cells is increased in vivo and in vitro when agents such as picrotoxin are administered^28, 29^, and hyperstimulating manipulations can exacerbate tau pathology^30^. Given that the EC is one of the first regions to be affected by overt tauopathy in the earliest stages of human AD^44^, our data showing that *APOE4* is associated with neuronal hyperactivity in this region could indicate that *APOE4* may accelerate the accumulation and propagation of Aβ or tau (or both), thereby accelerating the onset of AD in *APOE4* carriers. Intriguingly, EC hyperactivity has also been reported in non-demented adults with Down syndrome, another group at significant risk of developing AD^65^, suggesting that this may be a common mechanism among populations that are vulnerable to AD.

Functional imaging studies using fMRI^40, 66, 67^ or fluorodeoxyglucose positron emission tomography^68, 69^ have documented EC hypometabolism at various stages of AD, including a recent study from our lab^70^. Interestingly, APP metabolites such as Aβ have been shown to be synaptotoxic^71–73^, and tauopathy is known to correlate with cell loss^74^ and synaptic dysfunction^75^. As a decrease in CBV correlates with synaptic loss^76^, it is possible that the neurotoxic effects exerted by the overt accumulation of these pathological proteins could lead to the observed hypometabolism in AD. Given these observations, we hypothesize that *APOE4*-associated hyperactivity, particularly in the EC, may drive the accumulation of Aβ and tau, which would then lead to neurodegeneration and ultimately result in a hypoactive state once the disease process has reached a critical stage. In this way, the *APOE4*-associated hyperactivity reported here may act as a central mediator of AD pathogenesis among APOE4 carriers and may therefore represent a prime target for therapeutic intervention in AD.

## Methods

### Mice

Human *APOE*-targeted replacement mice were first developed by Sullivan et al.^37, 38^. All mice used in this study were treated in accordance with the National Institutes of Health Guide for the Care and Use of Laboratory Animals and approved by the Columbia University Medical Center and SUNY Medical Center Institutional Animal Care and Use Committee (IACUC). For the fMRI analysis, two groups of mice were utilized, one with a mean age of 8 months, and one with a mean age of 20 months. For the in vivo electrophysiology experiment, mice were aged to a mean age of 18 months. For metabolite profiling, mice possessed a mean age of 14.5 months. And for in vitro electrophysiology experiments, mice with a mean age of 20 months were utilized. All experiments were performed on male *APOE* mice.

### Mouse fMRI

A steady-state gadolinium enhancement approach was used to generate CBV maps, as previously described^40^. Briefly, images were acquired with a Bruker AVANCE 400WB spectrometer outfitted with an 89-mm bore, 9.4-T vertical Bruker magnet (Oxford Instruments), a 30-mm inner-diameter birdcage radio frequency coil, and a shielded gradient system (100 G/cm) before and 37.5 min after intraperitoneal injections of the contrast agent gadolinium (10 mmol/kg). Mice were anesthetized using a gaseous mixture of 30% O_2_, 70% N_2_, and isoflurane (3% volume for induction, 1.1–1.5% for maintenance at 1 liter per min air flow, via a nose cone). T2-weighted images were acquired perpendicular to the hippocampal long axis with a fast-spin echo acquisition (time to repeat, 2000 ms; time to echo, 70 ms; rapid acquisition and relaxation enhancement factor, 16; in plane resolution, 86 m; slice thickness, 500 m). Relative CBV maps were generated according to the formula CBV ∞ ∆R2 = ln (Spre/Spost) / TE; where TE = effective echo time; Spre = signal before contrast; Spost = signal contrast agent reaches steady state. Relative CBV was then derived from this image by normalizing its signal to the mean CBV value present in internal jugular vein.

For voxel-based analysis, image processing was conducted using custom bash and MATLAB scripts. Volumes were co-registered into a group-wise template space using an inverse-inconsistent diffeomorphic co-registration algorithm, as described previously^77^. Briefly, the co-registration incorporates a stationary vector field that smoothly warps images in their native coordinate space while conserving topology. Furthermore, each iteration of the co-registration was optimized by a Jacobian weighting term that encodes local distortions generated by the warp. This method yields robust and accurate alignment of images within their native coordinate space. The co-registration consisted of three steps. Anatomical images were first aligned to a randomly selected image in their data set using a linear transformation with 12 degrees of freedom. Next, the linearly aligned images were used to determine the optimal diffeomorphic transformation necessary to warp each image in the data set to an unbiased template image. Each iteration of the pairwise diffeomorphic registration between each image and the template generated a normalized warp with an average deformation of zero, ensuring that the template image generated was not biased by relative local volume loss or expansion between groups. Finally, the linear transformation and nonlinear warps were applied to corresponding CBV maps for each subject.

CBV maps were analyzed using a general linear model implemented in SPM8 (Wellcome Department of Imaging Neuroscience). Data were modeled in a factorial framework with genotype as the between subject factor. Individual genotypes were contrasted using Student’s *t* test. Results were height thresholded and corrected for multiple comparisons at the cluster level using a Monte-Carlo simulation run for 10,000 iterations to yield a cluster volume corrected to *p* < 0.05. Thresholded *t* maps from individual group comparisons were then overlaid onto their respective groupwise templates in cross-section using 3DSlicer (www.slicer.org) and displayed as maximum intensity projections over volume ray cast mesh models of the groupwise template and hippocampal formation, also generated in 3DSlicer. Mesh models of the whole-brain template were generated from manual segmentations of individual mice, which were transformed into template space by applying the non-linear warp. Individual segmentations of the hippocampal formation were transformed similarly. The hippocampal formation ROI was segmented using operational criteria described previously^45^. Specifically, this ROI included high-resolution segmentation of the DG, CA3, CA2, CA1, subiculum, pre-subiculum, para-subiculum, medial EC, and lateral EC.

### In vivo electrophysiology recordings

Custom-made, reusable 16-channel microdrives (Axona, UK) were constructed as described previously^46^. Tetrodes were created by twisting four 25 mm thick platinum-iridium wires (California wires) and heat bonding them. Four tetrodes were inserted into the inner cannula of the microdrive and connected to the wires of the microdrive. 1 day prior to surgery, the tetrodes were cut to an appropriate length and plated with a platinum solution until the impedance dropped to about 150 ohm. On the day of surgery, mice were anesthetized with isoflurane (1–2%) and monitored for depth of anesthesia before proceeding. Mice were then fixed within the stereotaxic frame with the use of zygomatic process cuff holders, after which an incision was made to expose the skull and about 3–4 jeweler’s screws were inserted into the skull to support the microdrive implant. An additional screw connected with wire was also inserted into the skull, which served as a ground/reference for LFP recordings. A 2 mm hole was made on the right hemisphere of the skull at 4.3 mm lateral and 3.4 mm posterior to bregma. Tetrodes were then lowered to about 2.5 mm from the surface of the brain (below dura). Dental cement was spread across the exposed skull and secured with the microdrive. Mice usually recovered within 24 h, after which the tetrodes were lowered and recording began. All recording depths reported are from the surface of the brain.

The mice explored a white box (45 × 45 cm) and underwent two recording sessions per day, with ≥4 h between sessions. Tetrode positions were moved not more than 100 µm at a time, and only after the last recording session of the day, allowing >12 h of stable electrode positioning prior to the next recording session. Neuronal signals from experimental mice were recorded using the Axona DacqUSB system. The signals were amplified 15,000–30,000 times and band pass-filtered between 0.8 and 6.7 kHz. LFPs were recorded from four channels of the electrodes. The LFP was amplified 15,000 times, low pass-filtered at 125 Hz, and sampled at 250 Hz. Notch filter was used to eliminate 60 Hz noise. The recording system tracked the position of the infrared LED on the head stage (sampling rate 50 Hz) by means of an overhead infrared video camera. Position data were speed-filtered, with only speeds of 5–20 cm/s used for LFP measurements. Tracking artifacts were removed by deleting samples greater than 100 cm/s and missing positions were interpolated with total durations <1 s, and then smoothing the path with a 21-sample boxcar window filter (400 ms; 10 samples on each side). For single unit recordings, a total of 155 excitatory neurons and 26 interneurons were detected at a depth of 3.1–3.3 mm in the nine mice used in this study. Putative excitatory cells were distinguished from putative interneurons based on waveform width (>300 µs), as described previously^48^. Investigators were blinded to genotype assignments during surgery, recordings, and single unit validation.

### Metabolite profiling

Mice were killed by cervical dislocation to maintain the brain environment, and individual brain regions were immediately removed and snap-frozen on dry ice. Tissues were stored at −80°C prior to extraction. Metabolite extraction was performed using a methyl tert-butyl ether (MTBE)/methanol extraction protocol modified from previous reports^78, 79^. Briefly, individual EC or PVC tissues were homogenized in 400 μl of ice-cold methanol using a bead mill homogenizer (TissueLyser II, Qiagen) at 25 beats per s, 2× for 45 s each. Following homogenization, samples were incubated in 1200 μl of MTBE for 1 h at room temperature to separate organic-soluble lipids from aqueous-soluble lipids and other small molecules. Finally, 360 μl of ultrapure water was added (for a final ratio of 3:1:0.9 MTBE:methanol:water) to resolve the two liquid phases, and the samples were centrifuged at 10,000×*g* for 10 min. The lower aqueous phase and the upper organic phase was collected from each sample and stored in separate tubes, and the remaining protein pellets were resuspended in 25 mM ammonium bicarbonate, pH 8, with 2.5% SDS. A BCA protein assay was performed on each protein fraction, and both the aqueous phase and organic phase were normalized to their protein concentration equivalent with 50% and 100% methanol, respectively. All samples were then stored at −80°C prior to analysis.

Metabolite profiling was performed using an Agilent Model 1200 liquid chromatography system coupled to an Agilent Model 6230 time-of-flight mass analyzer as described previously^49^. Metabolite separation was accomplished using aqueous normal phase gradient chromatography on a Diamond Hydride column (Microsolv), with mobile phase buffer A consisting of 50% isopropanol and 0.025% acetic acid, and mobile phase buffer B consisting of 90% acetonitrile and 5 mM ammonium acetate. Each aqueous phase sample was analyzed in both positive and negative ion detection mode for comprehensive coverage of metabolites with a mass of 50–1000 Da. Prior to analysis, it was observed that one APOE3/4 PVC sample possessed a chromatographic error and was removed from all future analysis.

Following MS analysis, the raw data was analyzed using Agilent Profinder (version B.06.00) and Mass Profiler Professional (version 12.0) software. Briefly, Profinder performs batch recursive feature extraction, which consists of a first-pass identification of features (ions) that possess a common elution profile (e.g., identical mass-to-charge (*m*/*z*) ratios and retention times), followed by a recursive analysis to re-mine each sample for the presence of features that were identified in at least one sample. Feature extraction was limited to the top 2000 metabolites per ion detection mode, with each metabolite possessing a minimum abundance of 600 ion counts. Each feature was also manually validated following extraction (with investigators blinded to genotype assignments during validation), and re-integration was performed when necessary. In addition to this untargeted feature extraction, we also used ProFinder to perform a targeted feature extraction, matching features against a proprietary database of 626 biologically relevant metabolites whose mass and retention times were previously determined using pure chemical standards. For this analysis, we allowed for a maximum mass deviation of 10 mDa and a retention time deviation of 1 min, and identified features were manually validated following extraction. For both untargeted and targeted feature extraction, Profinder analysis was followed up by multivariate and differential expression analysis using Mass Profiler Professional. Principle component analysis and hierarchical clustering analysis were used to examine data sets for common expression patterns, expected and unexpected clusters, and the presence of outlying samples. Following differential expression analysis, metabolites identified as differentially expressed were further validated for chromatographic and biological accuracy, and non-validated metabolites were removed.

### In vitro electrophysiological recordings

Mice were anesthetized with Ketamine/Xylazine (100/10 mg/kg). Mice were decapitated with an animal guillotine, and the brains were rapidly dissected out of the skull cavity. The cutting solution contained the following in mM: 130 potassium gluconate, 5 KCl, 20 HEPES acid, 25 glucose, 0.05 kynurenic acid, 0.05 EGTA-K, and pH equilibrated at 7.4 with KOH. Recordings were performed at an approximate position A/P −3.8 mm, M/L 3.0 mm, and D/V −5.6 mm (based on the C57Bl/6J mouse brain atlas http://www.mbl.org/atlas/atlas.php). The horizontal brain slices contained all the regions of the hippocampal formation: DG, CA3, CA1, subiculum, pre/para subiculum, lateral and medial EC and perirhinal cortices (this slice has an orientation that is similar to the middle hippocampus MRI slice used). After slicing, the tissue was placed in artificial CSF (aCSF) that contained the following in mM: 157 Na^+^, 136 Cl^−^, 2.5 K^+^, 1.6 Mg^2+^, 2 Ca^2+^, 26 HCO3^−^, and 11 d-glucose. Perfusion rate for aCSF during experiments was set at 1 ml/min with a peristaltic pump (Rabbit MiniPuls 2, Rainin Intruments Co.). Experiments were performed in an interface chamber (Fine Scientific Tools, Vancouver Canada). For recordings of sEFPs in the hippocampal formation, we used an extracellular aCSF containing 5 mM K^+^, which is known to reduce apparent synaptic failures and increase neuronal excitability, without promoting convulsant activity^54^. Slices were perfused with aCSF continuously bubbled with 95% O_2_/5% CO_2_, to maintain pH near 7.4 and the temperature was set at 34 °C. In all experiments, the slices were allowed to recover from the isolation procedure for at least 1.5 h before the beginning of the experiments. Borosilicate electrodes (1.5 mm outer diameter with filament, World Precision Instruments, Inc.) of 2–3 MΩ filled with 150 mM NaCl were used to measure LFPs. The stimulating electrode was connected to a stimulus isolation unit (Grass S88). An input–output relationship curve was obtained (basal synaptic transmission evaluation), and for the rest of the experiment, a stimulus evoking ~50–60% of the maximum field excitatory post-synaptic potential (fEPSP) response was selected. Extracellular recordings were performed using an Axoclamp 2B amplifier (Molecular Devices, Palo Alto, CA), filter (0.1 Hz–10 KHz using −6 dB/octave). Voltage signals were digitized and stored in a PC using a digidata 1200 A (Molecular Devices) for off line analysis. Data was collected for 20 min per regions per slice and analyzed using Neuromatic routines run in an Igor platform (Wavemetrics), with the threshold for events being a defection in amplitude 3 SD from noise and >20 ms in duration.

For the LTP induction, a stimulus as described above was used and a baseline was obtained (15 min with an inter-stimulus interval of 30 s). HFSs (3 trains of 100 pulses at 100 Hz, 10 seconds interval) were then applied, and responses were recorded for 50 min after tetanization and measured as fEPSP slope (10–90%), expressed as percentage of baseline. Basal fEPSP slopes, as well as the input–output relationships, were similar before stimulation, and they were normalized separately. To ensure that elicited fEPSP were indeed glutamatergic, at the end of each recording, NMDA- and AMPA-mediated currents were blocked with APV (30 μM) and NBQX (10 μM).

For the current clamp and NMDA-induced burst experiments, horizontal brain slices as described above were obtained. Pyramidal neurons (*n* = 6 cells per group) were selected for analysis. Glass pipettes were pulled from borosilicate glass (1.5 mm outer diameter with filament, World Precision Instruments, Inc). Pipette resistance was 4–5 MΩ. Intracellular solution was based on potassium gluconate (in mM): 166, MgCl_2_ 4.6, HEPES acid 10, ATP-Na 4, GTP-Na 0.4, and pH was balanced at 7.3–7.4 with KOH. Oregon Green 488 BAPTA-1 (OG1; Thermo Fischer Scientific) was used as a non-ratiometric calcium indicator, and added to the intracellular solution (final concentration of 50 µM). Series resistance was compensated, and access resistance (10–20 MΩ) was monitored regularly. Liquid junction potential was calculated and compensated for the holding potential. Signals were acquired using an amplifier Axopatch 200B (Molecular Devices), digitized with a Digidata 1550B (Molecular Devices), and processed with pClamp 10 (Molecular Devices). Signals were filtered at 5 kHz and digitized at 10 kHz. Neurons were discarded if the leak current was larger than 100 pA at voltage holding of −70 mV, or if the access resistance was larger than 20 MΩ. In the whole-cell configuration, pyramidal neurons were recorded in current clamp mode, and responses to current injection hyperpolarizing and depolarizing were used as indicated in each experiment. Neuronal firing was compared between the groups by injecting 250 pA for 4 s. For calcium imaging, an OptoLED system (Cairn, UK) was used as the light source, and excitation light was provided by a 470-nm LED. Light signals were captured by an iXon DV887 EM-CCD camera (Andor, USA). Software developed by Andor (Solis) was used with an image acquisition of 20 ms-frame (effective pixel size after ×60 objective = 266.7 nm; 1 × 1 binning). An in-house routine developed in an IGOR Pro programming environment (WaveMetrics, Lake Oswego, OR) was used for the analysis of calcium signals. After focal application of NMDA (1 mM, 200 ms) calcium signals were measured by calculating the average fluorescence in ROIs as a function of time. The size of the ROI was chosen as to just enclose the apical dendrite. Values are expressed as the percentage of change in fluorescence with respect to control, Δ*F*/*F*_0_ = 100 (*F*−*F*_0_)/(*F*_0_−*B*), where: *F* corresponds to the fluorescence signal from the ROI in the apical dendrite after the induction of the calcium transient at any given time; *F*_0_ corresponds to the average basal fluorescence signal previous to the induction of the calcium transients; and *B* is the average value at each time point of the background fluorescence from four regions of the imaged field that do not contain any part of the dye-filled cell. A glass pipette filled with NMDA and a pressure system (Picospritzer II, General Valve Corporation) positioned near the recorded neurons was used to induce fSLDs in cells kept in current clamp, with no current injection. fSLDs were blocked completed by APV 10 uM.

For mIPSC recordings, a new group of mice of the same age were anesthetized with Ketamine/Xylazine, and horizontal brain slices were obtained, as described above. All recordings were performed from layer II EC. In whole-cell configuration, pyramidal neurons (*n* = 7 cells from *APOE3* and *n* = 8 cells from *APOE4*) were recorded in voltage-clamp mode (Vh = −65 mV) in the presence of TTX (1 µM). A different internal solution (in mM: KCl 120, MgCl_2_ 4.6, EGTA 10, CaCl_2_ 1, HEPES acid 10, ATP-Na 4, GTP-Na 0.4) was used for these recordings. Additionally, AMPA receptors and NMDA receptors were blocked with bath applied NBQX (16 µM) and 2 APV (10 µM), respectively. At the end of the experiment, a GABA-A antagonist (picrotoxin, 200 µM) was used to confirm the recording of GABAergic synapses, as has been reported previously^80^. In every case, the synaptic currents were fully blocked by picrotoxin. The recorded mIPSCs were analyzed with Clampfit (Molecular Devices). Individual mIPSC events were detected with a template (threshold of 3.5) which was three times the SD from baseline noise and visually inspected to exclude artifacts. When data were pooled for this analysis, randomly selected 50 events were obtained from a continuous recording period of 3 min for each neuron. We have also included mean values in our analysis, as they more closely reflect the population distributions comprising our cumulative probability data. For all in vitro electrophysiology experiments, investigators were blinded to genotype assignment during recordings and analyses.

### Histological analysis

Mouse brains were removed after transcardial perfusion, and drop-fixed with 4% paraformaldehyde overnight, followed by cryoptrotection in 30% sucrose in PBS for 16 h. Free-floating brain sections from brains sectioned in the horizontal plane were used for immunohistochemistry using SuperPicTure^™^ polymer detection kit (Zymed, San Francisco, CA). Sections were washed with PBS for 10 min, and then treated with 3% H_2_O_2_ in PBS for 10 min. The sections were then transferred to a microfuge tube that contained primary antibody diluted in PBS containing 0.3% Triton and 5% normal serum, and incubated at 4 °C overnight on a rotator. After three washes with PBS-T (0.1% Tween 20), the sections were incubated for 10 min with HRP polymer conjugate. Following three washes with PBS-T, the immunoreactive material was visualized using DAB as a chromogen. The stained sections were mounted on slides and inspected by light microscopy. For murine tau detection, antibody CP13 (pS202) was utilized (a gift from Dr. P. Davies). Monoclonal antibody 4G8 was used to detect murine APP/Aβ.

### Statistical analysis

For each experiment, mouse numbers were selected to be similar to those used in previous experiments utilizing mouse fMRI^52, 70^, in vivo electrophysiology^30^, metabolite profiling^81, 82^, and in vitro electrophysiology^52^. For fMRI analysis, statistical analysis was performed using SPM8 software, as described above. For in vivo electrophysiological recordings, statistical analysis for LFP and single unit measurements was performed with Prism 6 (Graphpad) using an unpaired Student’s *t* test between groups with Welch’s correction. For metabolite profiling, differential expression analysis was performed using Mann–Whitney tests, with a Benjamini Hochberg correction applied to adjust for FDRs considering multiple group comparisons. For untargeted comparisons, reporting was restricted to those metabolites with an FDR-corrected *p* value of <0.05, while for targeted metabolite analyses, the window was widened to include metabolites with an uncorrected *p* value of <0.05. For in vitro electrophysiological recordings, statistical analysis for sEFP measurements was performed using non-parametric Mann–Whitney tests to generate *p* values. For LTP measurements, a repeated measures ANOVA with unequal variance was used to generate *F* and *p* values, and a Student’s *t* test with unequal variance was used to generate *t* and *p* values for the mean potentiation comparison. For current clamp and NMDA-induced burst experiments, we used one-way ANOVA for the statistical analysis. For mIPSC recordings, mIPSC amplitude, cumulative amplitudes, cumulative frequencies, instantaneous frequencies, and decay time constants were compared and analyzed (according to their distribution) using *t* test or Kolmogorov–Smirnov test (K–S) with the significance level set at *p* < 0.05.

### Data availability

The metabolomics data generated during the study are available through MetaboLights: http://www.ebi.ac.uk/metabolights/MTBLS530. All other data are available from the authors upon request.

## Electronic supplementary material

Supplementary Information
