# Supplementary material for: Neuronal hyperactivity due to loss of inhibitory tone in APOE4 mice lacking Alzheimer’s disease-like pathology
Source: Nat Commun. 2017 Nov 13;8:1464. doi: 10.1038/s41467-017-01444-0 (PMC5684208; doi:10.1038/s41467-017-01444-0)
Supplement: Supplementary file 1 — Supplementary Information [file 41467_2017_1444_MOESM1_ESM.pdf]

SUPPLEMENTAL INFORMATION

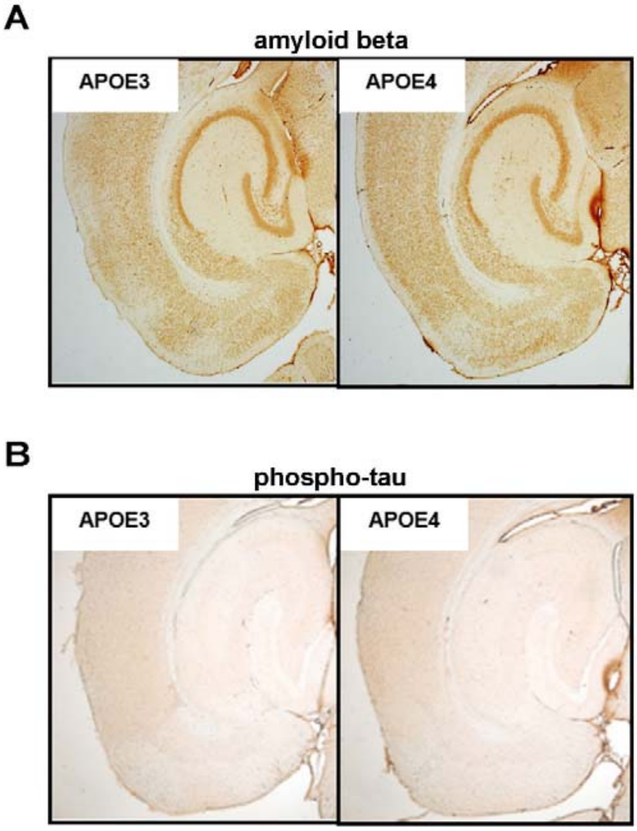

Supplementary Fig. 1. *APOE* mice show no AD pathology. Immunohistochemistry performed on brain sections from the *APOE* mice used during the fMRI study show no evidence of Aβ or tau pathology. (A) A postmortem hippocampal section of a 20 month old *APOE3* mouse or 20 month old *APOE4* mouse labeled with an antibody recognizing mouse Aβ (antibody 4G8) shows no histological evidence of amyloid pathology. (B) A postmortem hippocampal section of the same mice as A, labeled with an antibody recognizing phospho-tau (CP13), shows no histological evidence of tau pathology.

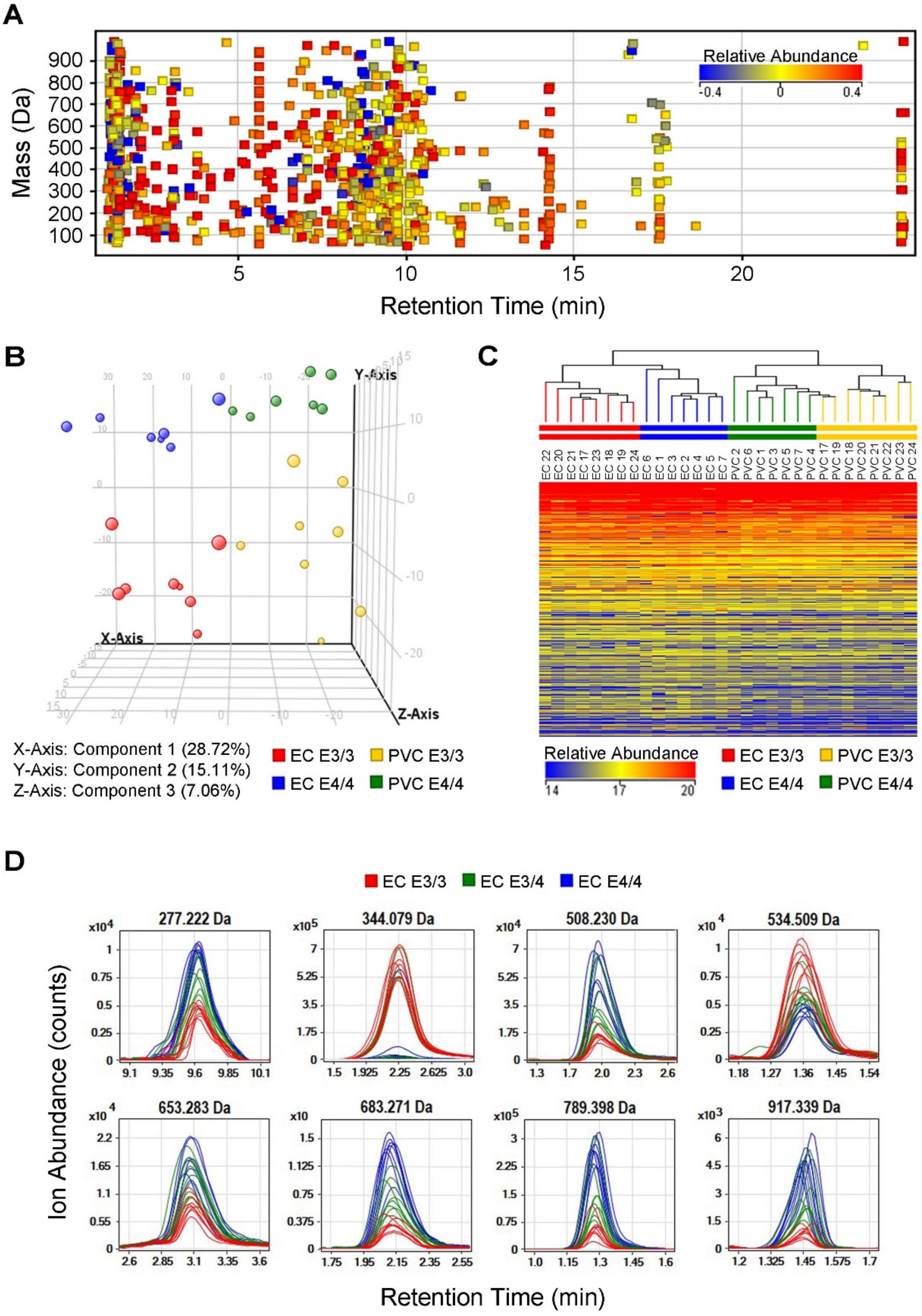

A
Mass (Da)
Retention Time (min)
Relative Abundance
B
X-Axis
Y-Axis
Z-Axis
X-Axis: Component 1 (28.72%)
Y-Axis: Component 2 (15.11%)
Z-Axis: Component 3 (7.06%)
EC E3/3
PVC E3/3
EC E4/4
PVC E4/4
C
Relative Abundance
EC E3/3
PVC E3/3
EC E4/4
PVC E4/4
D
EC E3/3
EC E3/4
EC E4/4
277.222 Da
344.079 Da
508.230 Da
534.509 Da
653.283 Da
683.271 Da
789.398 Da
917.339 Da
Ion Abundance (counts)
Retention Time (min)

Supplementary Fig. 2. Small-molecule expression reveals genotypic and regional differences in aged APOE mice. Untargeted metabolite profiling was performed on small-molecule metabolites extracted from the EC and PVC of aged *APOE* mice (14-15 months old; 8 *APOE3/3*, 9 *APOE3/4* and 7 *APOE4/4* males). (A) A Mass vs. Retention Time plot depicting all ions discovered in positive ion detection mode using untargeted analysis. (B) Principle component analysis on untargeted metabolites discovered in positive ion detection mode. Each sphere represents a single sample. (C) Hierarchical clustering analysis on untargeted metabolites discovered in positive ion detection mode. Samples names are listed on top, with each line in the heat map representing the relative abundance of a single metabolite. (D) Chromatograms of eight metabolites detected in positive ion detection mode in the EC.

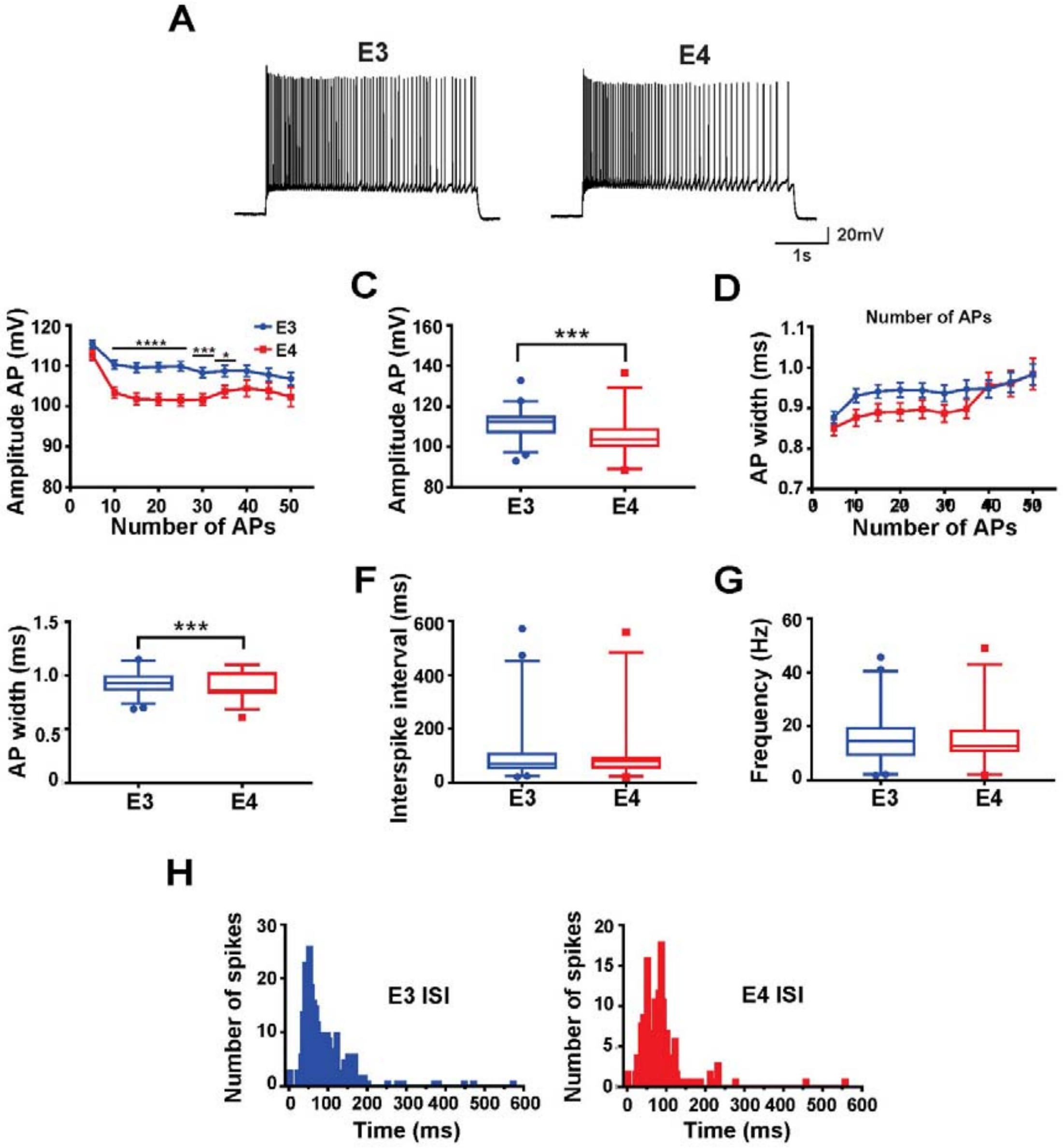

Supplementary Fig. 3. ***APOE4*** **expression is associated with decreased amplitude and width of action potentials in layer II EC pyramidal cells**. In vitro electrophysiology was performed using the patch-clamp whole-cell modality on pyramidal cells (n=6 cells per group) in the layer II EC of aged *APOE3* and *APOE4* mice (mean age = 20 months). (A) Representative traces of pyramidal neurons of *APOE3* and *APOE4* mice. Neurons were kept in current-clamp, and current was injected (0.3 nA, 4 s) in order to induce a train of action potentials (APs). (B) The amplitude of the AP was plotted after induction

of an AP train in *APOE3* and *APOE4* mice (****p<0.001; ***p=0.001; *p=0.015). (C) Box plot bars showing (CI 1-99%) grouped AP amplitudes from B (ANOVA; $F_{(1, 471)}$=88.784, p<0.001). (D) AP width after induction of AP train. (E) Box-plot bars of AP width from *APOE3* and *APOE4* mice (ANOVA; $F_{(1, 471)}$=19.777, p<0.001). (F) Histograms of the interspike-interval (ISI) from *APOE3* (top) and *APOE4* (bottom). (G) Box-plot bars of frequencies of APs from layer II EC neurons from *APOE3* and *APOE4* mice (ANOVA; $F_{(1, 460)}$=0.142, p=0.706). (H) Box-plot bars of ISI of APs from layer II EC neurons from *APOE3* and *APOE4* mice (ANOVA; $F_{(1, 460)}$=0.061, p=0.806).

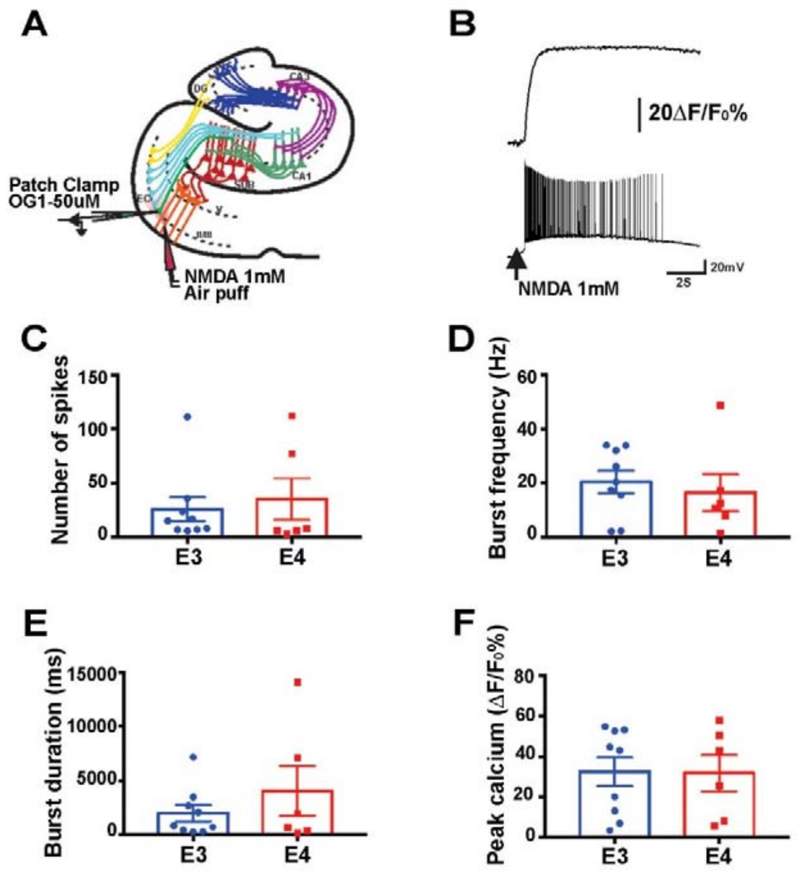

Supplementary Fig. 4. *APOE4* expression is not associated with increased NMDA-receptor firing in EC layer II pyramidal cells. NMDA-induced bursts were recorded using the patch-clamp whole-cell modality on pyramidal cells (n= 6 cells per group) in the layer II EC of aged *APOE3* and *APOE4* mice (mean age = 20 months). (A) Representation of the experiment design: a hippocampal horizontal slice is depicted with the principal neurons and projections in the different subregions. Layer II EC neurons were recorded with simultaneous calcium imaging (pipette solution contains, Oregon Green 1 BAPTA, 50 µM). A second glass pipette with NMDA (1mM) was placed close to the recording neuron, and an air-pressure system produced a quick release of NMDA (1 puff, 200 ms, 7 psi). (B) A representative trace of simultaneous current-clamp and calcium imaging recordings performed in layer II EC pyramidal neurons. **Top.** Changes in calcium fluorescence ($\Delta F/F_0\%$) normalized to the baseline pre-stimulus. **Bottom.** Current-clamp trace after a stimulation with the NMDA pipette (arrow). (C) Number of spikes induced

after the stimulus with the NMDA pipette (ANOVA; $F_{(1, 13)}$=0.218, p=0.649). (D) Burst frequency of induced APs (ANOVA; $F_{(1, 13)}$=0.270, p=0.612). (E) Burst duration of induced APs (Welch ANOVA; $F_{(1, 6.113)}$=0.735, p=0.424). (F) Peak calcium (ANOVA; $F_{(1, 13)}$=0.003, p=0.96).

Supplementary Table 1. Differentially expressed untargeted metabolites from the EC of APOE3 vs. E4 mice

Mass (Da) @ Retention Time (min)	Regulation in E4/4	Fold Change	p-value	FDR	Detection Mode
**386.186@1.277**	up	3.25	0.001	0.012	positive
**191.151@9.700**	up	1.48	0.001	0.012	positive
**295.251@1.347**	down	1.31	0.001	0.012	positive
**403.209@1.277**	up	2.72	0.001	0.012	positive
**772.707@1.277**	up	3.55	0.001	0.012	positive
**279.254@1.379**	down	1.38	0.001	0.012	positive
**789.398@1.275**	up	6.27	0.001	0.012	positive
**173.140@9.690**	up	1.66	0.001	0.012	positive
**281.270@1.379**	down	1.55	0.001	0.012	positive
**681.437@1.328**	up	2.26	0.001	0.012	positive
**330.121@1.276**	up	2.78	0.001	0.012	positive
**667.456@1.355**	up	2.25	0.001	0.012	positive
**665.441@1.363**	up	2.43	0.001	0.012	positive
**297.264@1.344**	down	1.33	0.001	0.012	positive
**556.448@1.344**	down	1.28	0.001	0.012	positive
**387.356@1.276**	up	3.75	0.001	0.012	positive
**683.449@1.329**	up	2.05	0.001	0.012	positive
**97.088@9.700**	up	1.47	0.001	0.012	positive
**472.270@1.247**	up	6.65	0.001	0.012	positive
**472.280@1.211**	down	13.97	0.001	0.012	positive
**136.073@2.354**	up	4.07	0.001	0.012	positive
**508.230@2.083**	up	3.37	0.001	0.012	positive
**386.779@1.279**	up	3.85	0.001	0.012	positive
**467.184@1.436**	up	2.49	0.001	0.012	positive
**701.462@1.432**	up	4.04	0.001	0.012	positive
**200.046@5.028**	up	1.98	0.001	0.012	positive
**742.247@1.458**	up	1.87	0.001	0.012	positive
**536.526@1.393**	down	1.78	0.001	0.012	positive
**251.224@1.397**	down	1.24	0.001	0.012	positive
**900.685@1.443**	up	2.73	0.001	0.012	positive
**155.130@9.692**	up	1.63	0.001	0.012	positive
**640.135@10.140**	up	1.74	0.001	0.012	positive
**697.432@1.323**	up	2.25	0.001	0.012	positive
**180.019@1.418**	up	1.84	0.001	0.012	positive
**699.446@1.385**	up	2.89	0.001	0.012	positive

**574.210@9.700**	up	1.68	0.001	0.012	positive
**269.238@1.355**	down	1.41	0.001	0.012	positive
**580.217@1.283**	up	2.48	0.001	0.012	positive
**667.457@1.355**	up	2.25	0.001	0.012	positive
**574.318@9.703**	up	1.66	0.001	0.012	positive
**900.690@1.443**	up	2.40	0.001	0.012	positive
**772.944@1.276**	up	12.77	0.001	0.012	positive
**506.475@1.386**	down	1.54	0.001	0.012	positive
**118.063@2.355**	up	3.65	0.001	0.012	positive
**403.206@1.539**	up	4.48	0.001	0.012	positive
**653.402@1.344**	up	3.62	0.001	0.012	positive
**262.227@1.379**	down	1.35	0.001	0.012	positive
**403.381@1.274**	up	3.49	0.001	0.012	positive
**80.062@1.379**	down	1.29	0.001	0.012	positive
**789.631@1.270**	up	9.38	0.001	0.012	positive
**261.192@9.629**	up	1.35	0.001	0.012	positive
**794.355@1.275**	up	5.83	0.001	0.012	positive
**246.221@9.299**	up	1.87	0.001	0.012	positive
**683.271@2.120**	up	5.47	0.001	0.012	positive
**560.518@1.380**	down	1.97	0.001	0.012	positive
**695.423@1.342**	up	2.59	0.001	0.012	positive
**710.620@1.386**	down	1.70	0.001	0.012	positive
**776.853@1.271**	up	4.02	0.001	0.012	positive
**655.428@1.334**	up	2.08	0.001	0.012	positive
**990.823@1.282**	up	2.22	0.001	0.012	positive
**990.829@1.282**	up	2.56	0.001	0.012	positive
**576.823@9.699**	up	1.58	0.001	0.012	positive
**277.222@9.631**	up	2.85	0.001	0.012	positive
**889.205@1.346**	down	1.43	0.001	0.012	positive
**566.508@1.426**	down	1.43	0.001	0.012	positive
**886.688@1.341**	down	1.45	0.001	0.012	positive
**854.355@1.441**	up	8.56	0.001	0.012	positive
**858.465@1.285**	up	10.13	0.001	0.012	positive
**265.227@1.385**	down	1.60	0.001	0.012	positive
**519.763@9.689**	up	1.79	0.001	0.012	positive
**120.089@1.368**	down	1.22	0.001	0.012	positive
**544.316@1.208**	up	2.43	0.001	0.012	positive
**333.213@1.354**	down	3.14	0.001	0.012	positive
**266.206@9.705**	up	3.73	0.001	0.012	positive
**307.198@9.729**	up	1.72	0.001	0.012	positive
**637.414@1.375**	up	3.77	0.001	0.012	positive

**636.165@1.273**	up	2.33	0.001	0.012	positive
**774.084@1.278**	up	6.45	0.001	0.012	positive
**508.493@1.369**	down	2.16	0.001	0.012	positive
**534.509@1.370**	down	2.18	0.001	0.012	positive
**241.213@1.375**	down	1.64	0.001	0.012	positive
**655.422@1.333**	up	2.05	0.001	0.012	positive
**323.273@1.334**	down	1.74	0.001	0.012	positive
**533.252@1.445**	up	8.86	0.001	0.012	positive
**917.339@1.443**	up	6.10	0.001	0.012	positive
**789.735@1.279**	up	17.67	0.001	0.012	positive
**629.372@1.971**	up	9.18	0.001	0.012	positive
**576.958@9.702**	up	1.63	0.001	0.012	positive
**274.063@1.281**	up	2.84	0.001	0.012	positive
**667.675@1.356**	up	2.72	0.001	0.012	positive
**731.430@1.422**	up	2.26	0.001	0.012	positive
**677.407@1.334**	up	2.66	0.001	0.012	positive
**713.422@1.350**	up	2.26	0.001	0.012	positive
**729.425@1.402**	up	2.67	0.001	0.012	positive
**316.093@2.535**	up	3.74	0.001	0.012	positive
**562.939@1.379**	down	1.78	0.001	0.012	positive
**660.673@1.274**	up	8.95	0.001	0.012	positive
**442.254@1.200**	up	5.47	0.001	0.012	positive
**807.153@1.274**	up	2.96	0.001	0.012	positive
**183.155@1.382**	down	1.90	0.001	0.012	positive
**934.369@1.435**	up	2.23	0.001	0.012	positive
**681.748@1.321**	up	2.71	0.001	0.012	positive
**657.447@1.374**	up	3.02	0.001	0.012	positive
**797.387@1.282**	up	14.72	0.001	0.012	positive
**731.437@1.422**	up	2.14	0.001	0.012	positive
**403.447@1.275**	up	4.17	0.001	0.012	positive
**627.413@1.356**	up	5.36	0.001	0.012	positive
**429.395@7.778**	up	2.58	0.001	0.012	positive
**684.237@1.451**	up	2.08	0.001	0.012	positive
**627.415@1.363**	up	4.08	0.001	0.012	positive
**485.278@9.312**	up	1.86	0.001	0.012	positive
**807.152@1.274**	up	3.31	0.001	0.012	positive
**700.137@1.418**	up	1.80	0.001	0.012	positive
**229.233@8.620**	up	1.24	0.001	0.012	positive
**178.166@1.379**	down	1.70	0.001	0.012	positive
**385.281@1.410**	down	1.56	0.001	0.012	positive
**422.163@1.266**	up	3.10	0.001	0.012	negative

**424.161@1.266**	up	3.19	0.001	0.012	negative
**446.206@1.268**	up	4.25	0.001	0.012	negative
**200.044@5.046**	up	1.62	0.001	0.012	negative
**450.158@1.415**	up	2.17	0.001	0.012	negative
**492.135@5.046**	up	1.66	0.001	0.012	negative
**742.247@1.458**	up	1.86	0.001	0.012	negative
**424.336@1.266**	up	4.37	0.001	0.012	negative
**449.181@1.261**	up	3.08	0.001	0.012	negative
**404.196@1.422**	up	4.10	0.001	0.012	negative
**264.014@7.119**	up	1.81	0.001	0.012	negative
**446.206@1.262**	up	4.48	0.001	0.012	negative
**191.916@5.047**	up	1.59	0.001	0.012	negative
**422.783@1.268**	up	5.40	0.001	0.012	negative
**334.046@10.373**	up	4.27	0.001	0.012	negative
**200.168@5.048**	up	1.66	0.001	0.012	negative
**514.093@1.270**	up	2.60	0.001	0.012	negative
**450.338@1.416**	up	2.55	0.001	0.012	negative
**286.119@4.311**	up	4.87	0.001	0.012	negative
**416.028@10.351**	up	4.10	0.001	0.012	negative
**333.238@1.351**	down	1.44	0.001	0.012	negative
**200.044@7.119**	up	1.56	0.001	0.012	negative
**374.073@10.325**	up	4.61	0.001	0.012	negative
**356.174@1.269**	up	2.62	0.001	0.012	negative
**424.780@1.260**	up	5.60	0.001	0.012	negative
**331.228@1.331**	down	1.42	0.001	0.012	negative
**434.038@10.351**	up	4.78	0.001	0.012	negative
**374.010@5.049**	up	1.50	0.001	0.012	negative
**850.822@1.268**	up	5.98	0.001	0.012	negative
**696.289@1.450**	up	4.71	0.001	0.012	negative
**556.106@7.113**	up	1.81	0.001	0.012	negative
**341.995@7.123**	up	1.61	0.001	0.012	negative
**235.997@2.353**	up	5.60	0.001	0.012	negative
**426.167@1.265**	up	3.36	0.001	0.012	negative
**349.234@1.379**	down	1.25	0.001	0.012	negative
**468.169@4.330**	up	2.16	0.001	0.012	negative
**196.090@2.343**	up	4.98	0.001	0.012	negative
**450.410@1.416**	up	3.20	0.001	0.012	negative
**191.917@7.124**	up	1.74	0.001	0.012	negative
**318.104@6.006**	up	2.97	0.001	0.012	negative
**386.186@1.269**	up	3.57	0.001	0.012	negative
**304.018@5.064**	up	1.36	0.001	0.012	negative

**432.198@1.265**	up	10.65	0.001	0.012	negative
**512.185@1.267**	up	1.62	0.001	0.012	negative
**254.132@1.229**	up	1.94	0.001	0.012	negative
**614.181@1.414**	up	2.04	0.001	0.012	negative
**610.197@6.008**	up	7.45	0.001	0.012	negative
**578.207@4.303**	up	7.30	0.001	0.012	negative
**742.476@1.460**	up	2.04	0.001	0.012	negative
**500.121@5.060**	up	1.46	0.001	0.012	negative
**594.088@4.925**	up	1.96	0.001	0.012	negative
**596.221@1.432**	up	4.26	0.001	0.012	negative
**490.203@1.294**	up	5.00	0.001	0.012	negative
**596.108@5.056**	up	1.61	0.001	0.012	negative
**586.201@1.249**	up	2.04	0.001	0.012	negative
**898.728@1.261**	up	3.62	0.001	0.012	negative
**218.013@2.383**	up	1.89	0.001	0.012	negative
**492.135@5.042**	up	1.68	0.001	0.012	negative
**336.074@4.992**	up	2.22	0.001	0.012	negative
**666.219@7.821**	up	2.22	0.001	0.012	negative
**614.182@1.414**	up	2.03	0.001	0.012	negative
**388.099@5.039**	up	1.49	0.001	0.012	negative
**382.075@7.279**	up	2.29	0.001	0.012	negative
**376.008@5.046**	up	1.50	0.001	0.012	negative
**386.041@4.301**	up	3.56	0.001	0.012	negative
**492.396@5.042**	up	3.35	0.001	0.012	negative
**431.105@5.042**	up	1.53	0.001	0.012	negative
**420.336@2.339**	up	17.11	0.001	0.012	negative
**510.233@1.225**	up	4.37	0.001	0.012	negative
**530.198@1.419**	up	6.40	0.001	0.012	negative
**451.990@5.046**	up	1.83	0.001	0.012	negative
**504.167@7.031**	up	2.00	0.001	0.012	negative
**642.203@6.955**	up	1.67	0.001	0.012	negative
**476.215@2.809**	up	4.94	0.001	0.012	negative
**192.119@5.045**	up	1.72	0.001	0.012	negative
**419.973@7.156**	up	1.51	0.001	0.012	negative
**764.188@7.811**	up	2.57	0.001	0.012	negative
**617.177@1.422**	up	2.56	0.001	0.012	negative
**184.050@4.293**	up	1.97	0.001	0.012	negative
**141.948@10.350**	up	16.89	0.001	0.012	negative
**780.203@1.449**	up	2.53	0.001	0.012	negative
**633.186@7.035**	up	2.44	0.001	0.012	negative
**596.179@2.361**	up	4.24	0.001	0.012	negative

**150.021@7.323**	down	4.38	0.001	0.012	negative
**795.160@1.430**	up	3.81	0.001	0.012	negative
**258.131@1.961**	up	2.37	0.001	0.012	negative
**828.275@8.413**	up	3.07	0.001	0.012	negative
**267.221@1.367**	down	1.26	0.002	0.016	positive
**255.254@1.404**	down	1.33	0.002	0.016	positive
**653.283@3.085**	up	2.11	0.002	0.016	positive
**574.706@9.701**	up	1.73	0.002	0.016	positive
**562.834@1.379**	down	1.55	0.002	0.016	positive
**886.824@1.347**	down	1.56	0.002	0.016	positive
**550.538@1.403**	down	1.72	0.002	0.016	positive
**371.174@8.148**	up	2.87	0.002	0.016	positive
**70.076@1.359**	down	1.37	0.002	0.016	positive
**800.543@1.198**	up	1.65	0.002	0.016	positive
**679.424@1.321**	up	1.27	0.002	0.016	positive
**551.281@1.285**	up	1.97	0.002	0.016	positive
**972.323@7.826**	up	3.06	0.002	0.016	positive
**802.076@1.364**	down	1.36	0.002	0.016	positive
**727.444@1.300**	up	1.50	0.002	0.016	positive
**459.226@7.870**	up	1.71	0.002	0.016	positive
**242.022@6.421**	up	1.96	0.002	0.018	negative
**534.110@6.421**	up	2.12	0.002	0.018	negative
**246.009@7.121**	up	2.37	0.002	0.018	negative
**524.092@1.410**	up	1.74	0.002	0.018	negative
**478.069@5.049**	up	3.98	0.002	0.018	negative
**250.219@1.350**	down	1.33	0.003	0.021	positive
**461.292@1.408**	up	1.38	0.003	0.021	positive
**717.565@1.398**	down	1.34	0.003	0.021	positive
**232.216@1.356**	down	1.23	0.003	0.021	positive
**260.210@1.362**	down	1.23	0.003	0.021	positive
**153.108@1.392**	down	1.38	0.003	0.021	positive
**465.170@1.438**	up	1.71	0.003	0.021	positive
**406.290@1.218**	down	1.53	0.003	0.021	positive
**521.198@7.040**	up	1.98	0.003	0.021	positive
**283.173@1.832**	up	7.14	0.003	0.021	positive
**665.662@1.360**	up	3.05	0.003	0.021	positive
**557.309@8.349**	up	1.93	0.003	0.021	positive
**835.103@1.346**	down	1.31	0.003	0.021	positive
**972.327@7.043**	up	2.69	0.003	0.021	positive
**622.794@1.402**	down	1.28	0.003	0.021	positive
**154.006@3.792**	up	1.31	0.003	0.024	negative

**335.239@1.357**	down	1.27	0.003	0.024	negative
**611.172@6.833**	up	1.82	0.003	0.024	negative
**104.044@2.228**	up	1.31	0.003	0.024	negative
**58.042@7.453**	up	1.22	0.003	0.024	negative
**321.212@1.368**	up	2.89	0.003	0.024	negative
**478.073@5.044**	up	4.19	0.003	0.024	negative
**482.181@1.290**	up	2.02	0.003	0.024	negative
**438.147@1.302**	down	1.65	0.003	0.024	negative
**749.941@1.160**	up	1.25	0.004	0.028	positive
**344.079@2.194**	down	17.74	0.004	0.028	positive
**327.199@2.027**	up	14.61	0.004	0.028	positive
**152.115@1.352**	down	1.36	0.004	0.028	positive
**574.604@9.700**	up	1.66	0.004	0.028	positive
**213.161@1.398**	down	1.69	0.004	0.028	positive
**118.064@5.030**	up	1.61	0.004	0.028	positive
**359.192@8.408**	up	1.86	0.004	0.028	positive
**463.073@10.792**	up	1.48	0.004	0.028	positive
**274.187@1.393**	down	1.27	0.004	0.028	positive
**448.144@1.438**	up	1.66	0.004	0.028	positive
**603.844@17.681**	down	1.17	0.004	0.028	positive
**118.026@1.376**	down	1.42	0.004	0.032	negative
**236.051@1.376**	down	1.37	0.004	0.032	negative
**464.101@10.288**	down	1.63	0.004	0.032	negative
**434.118@6.584**	up	1.33	0.004	0.032	negative
**612.175@1.435**	up	1.95	0.004	0.032	negative
**278.035@5.046**	up	1.81	0.004	0.032	negative
**617.173@1.417**	up	2.04	0.004	0.032	negative
**390.031@7.359**	down	4.37	0.005	0.043	negative
**194.115@2.535**	up	1.82	0.005	0.036	positive
**156.159@24.812**	up	1.62	0.005	0.036	positive
**150.088@2.295**	up	1.75	0.005	0.036	positive
**250.975@1.161**	up	1.22	0.005	0.036	positive
**386.970@1.279**	up	2.56	0.005	0.036	positive
**774.844@1.278**	up	1.80	0.005	0.036	positive
**422.287@2.533**	up	1.72	0.005	0.036	positive
**141.147@9.405**	up	1.49	0.005	0.036	positive
**241.219@1.382**	down	1.36	0.005	0.036	positive
**582.704@2.535**	up	1.76	0.005	0.036	positive
**612.477@1.440**	down	1.41	0.005	0.036	positive
**116.061@1.626**	up	2.42	0.005	0.036	positive
**618.595@1.554**	down	2.74	0.005	0.036	positive

**78.047@1.374**	down	1.51	0.005	0.036	positive
**150.135@1.369**	down	1.34	0.005	0.036	positive
**515.301@5.145**	up	5.91	0.005	0.036	positive
**90.030@7.680**	up	1.15	0.005	0.043	negative
**419.152@5.090**	up	1.37	0.005	0.043	negative
**500.174@1.273**	up	3.96	0.005	0.043	negative
**512.858@1.421**	down	1.41	0.005	0.043	negative
**523.049@6.882**	up	1.15	0.005	0.043	negative
**773.224@7.256**	up	2.02	0.005	0.043	negative
**511.103@9.165**	up	1.34	0.005	0.043	negative
**619.090@7.472**	up	2.33	0.005	0.043	negative
**238.142@2.995**	up	1.87	0.008	0.048	positive
**293.219@1.416**	down	1.21	0.008	0.048	positive
**277.234@1.439**	down	1.18	0.008	0.048	positive
**88.053@2.530**	up	1.98	0.008	0.048	positive
**311.245@1.415**	down	1.19	0.008	0.048	positive
**817.417@1.268**	up	1.94	0.008	0.048	positive
**82.044@1.285**	up	1.59	0.008	0.048	positive
**269.158@7.136**	up	2.87	0.008	0.048	positive
**714.828@2.990**	up	2.05	0.008	0.048	positive
**626.818@1.407**	down	1.21	0.008	0.048	positive
**443.326@1.386**	down	1.79	0.008	0.048	positive
**765.915@2.993**	up	2.77	0.008	0.048	positive

Supplementary Table 2. Differentially expressed untargeted metabolites from the PVC of APOE3 vs. E4 mice

Mass (Da) @ Retention Time (min)	Regulation in E4/4	Fold Change	p-value	FDR	Detection Mode
**386.186@1.277**	up	3.11	0.001	0.014	positive
**191.151@9.700**	up	1.51	0.001	0.014	positive
**403.209@1.277**	up	2.64	0.001	0.014	positive
**772.707@1.277**	up	3.55	0.001	0.014	positive
**279.254@1.379**	down	1.37	0.001	0.014	positive
**789.398@1.275**	up	6.08	0.001	0.014	positive
**173.140@9.690**	up	1.64	0.001	0.014	positive
**281.270@1.379**	down	1.43	0.001	0.014	positive
**681.437@1.328**	up	2.29	0.001	0.014	positive
**330.121@1.276**	up	2.60	0.001	0.014	positive
**667.456@1.355**	up	2.10	0.001	0.014	positive
**665.441@1.363**	up	2.15	0.001	0.014	positive
**297.264@1.344**	down	1.30	0.001	0.014	positive
**387.356@1.276**	up	3.69	0.001	0.014	positive
**683.449@1.329**	up	2.02	0.001	0.014	positive
**97.088@9.700**	up	1.51	0.001	0.014	positive
**472.270@1.247**	up	20.46	0.001	0.014	positive
**810.327@1.275**	up	5.15	0.001	0.014	positive
**472.280@1.211**	down	12.38	0.001	0.014	positive
**255.254@1.404**	down	1.27	0.001	0.014	positive
**136.073@2.354**	up	2.90	0.001	0.014	positive
**508.230@2.083**	up	3.89	0.001	0.014	positive
**386.779@1.279**	up	4.48	0.001	0.014	positive
**467.184@1.436**	up	2.81	0.001	0.014	positive
**424.141@1.280**	up	2.13	0.001	0.014	positive
**701.462@1.432**	up	3.64	0.001	0.014	positive
**174.008@1.273**	up	1.94	0.001	0.014	positive
**536.526@1.393**	down	1.59	0.001	0.014	positive
**251.224@1.397**	down	1.28	0.001	0.014	positive
**900.685@1.443**	up	8.45	0.001	0.014	positive
**155.130@9.692**	up	1.60	0.001	0.014	positive
**697.432@1.323**	up	2.17	0.001	0.014	positive
**180.019@1.418**	up	1.64	0.001	0.014	positive
**699.446@1.385**	up	2.57	0.001	0.014	positive
**574.210@9.700**	up	1.74	0.001	0.014	positive

**269.238@1.355**	down	1.36	0.001	0.014	positive
**580.217@1.283**	up	2.50	0.001	0.014	positive
**250.975@1.161**	up	1.14	0.001	0.014	positive
**667.457@1.355**	up	2.10	0.001	0.014	positive
**574.318@9.703**	up	1.68	0.001	0.014	positive
**900.690@1.443**	up	5.28	0.001	0.014	positive
**772.944@1.276**	up	10.49	0.001	0.014	positive
**506.475@1.386**	down	1.51	0.001	0.014	positive
**118.063@2.355**	up	2.75	0.001	0.014	positive
**262.227@1.379**	down	1.29	0.001	0.014	positive
**403.381@1.274**	up	3.62	0.001	0.014	positive
**789.631@1.270**	up	5.61	0.001	0.014	positive
**246.221@9.299**	up	1.76	0.001	0.014	positive
**683.271@2.120**	up	4.25	0.001	0.014	positive
**560.518@1.380**	down	1.96	0.001	0.014	positive
**695.423@1.342**	up	1.72	0.001	0.014	positive
**710.620@1.386**	down	2.26	0.001	0.014	positive
**776.853@1.271**	up	4.51	0.001	0.014	positive
**655.428@1.334**	up	1.81	0.001	0.014	positive
**990.823@1.282**	up	2.14	0.001	0.014	positive
**574.706@9.701**	up	1.70	0.001	0.014	positive
**990.829@1.282**	up	2.29	0.001	0.014	positive
**576.823@9.699**	up	1.61	0.001	0.014	positive
**277.222@9.631**	up	2.74	0.001	0.014	positive
**566.508@1.426**	down	1.53	0.001	0.014	positive
**886.688@1.341**	down	1.40	0.001	0.014	positive
**854.355@1.441**	up	6.49	0.001	0.014	positive
**858.465@1.285**	up	10.18	0.001	0.014	positive
**562.834@1.379**	down	1.54	0.001	0.014	positive
**544.316@1.208**	up	2.70	0.001	0.014	positive
**266.206@9.705**	up	2.65	0.001	0.014	positive
**636.165@1.273**	up	2.09	0.001	0.014	positive
**241.219@1.382**	down	1.27	0.001	0.014	positive
**774.084@1.278**	up	7.49	0.001	0.014	positive
**508.493@1.369**	down	2.10	0.001	0.014	positive
**534.509@1.370**	down	2.07	0.001	0.014	positive
**655.422@1.333**	up	1.80	0.001	0.014	positive
**917.339@1.443**	up	8.14	0.001	0.014	positive
**789.735@1.279**	up	14.76	0.001	0.014	positive
**723.519@1.621**	up	3.58	0.001	0.014	positive
**576.958@9.702**	up	1.68	0.001	0.014	positive

**274.063@1.281**	up	3.15	0.001	0.014	positive
**800.543@1.198**	up	1.72	0.001	0.014	positive
**713.422@1.350**	up	2.12	0.001	0.014	positive
**729.425@1.402**	up	2.37	0.001	0.014	positive
**316.093@2.535**	up	2.96	0.001	0.014	positive
**679.424@1.321**	up	1.38	0.001	0.014	positive
**562.939@1.379**	down	1.69	0.001	0.014	positive
**660.673@1.274**	up	11.80	0.001	0.014	positive
**442.254@1.200**	up	3.63	0.001	0.014	positive
**807.153@1.274**	up	4.24	0.001	0.014	positive
**934.369@1.435**	up	3.52	0.001	0.014	positive
**938.270@1.439**	up	5.64	0.001	0.014	positive
**681.748@1.321**	up	3.15	0.001	0.014	positive
**146.105@1.305**	up	1.42	0.001	0.014	positive
**797.387@1.282**	up	13.54	0.001	0.014	positive
**731.437@1.422**	up	2.44	0.001	0.014	positive
**403.447@1.275**	up	3.55	0.001	0.014	positive
**627.413@1.356**	up	4.23	0.001	0.014	positive
**429.395@7.778**	up	3.77	0.001	0.014	positive
**485.278@9.312**	up	1.88	0.001	0.014	positive
**745.417@1.429**	up	2.29	0.001	0.014	positive
**807.152@1.274**	up	4.64	0.001	0.014	positive
**203.114@13.469**	up	1.40	0.002	0.018	positive
**742.247@1.458**	up	2.34	0.002	0.018	positive
**84.021@13.470**	up	1.36	0.002	0.018	positive
**261.192@9.629**	up	1.49	0.002	0.018	positive
**241.270@8.482**	down	9.41	0.002	0.018	positive
**886.824@1.347**	down	1.31	0.002	0.018	positive
**369.296@1.310**	down	1.97	0.002	0.018	positive
**203.238@13.469**	up	1.48	0.002	0.018	positive
**241.213@1.375**	down	1.33	0.002	0.018	positive
**550.538@1.403**	down	1.39	0.002	0.018	positive
**371.174@8.148**	up	2.81	0.002	0.018	positive
**592.920@1.348**	down	1.44	0.002	0.018	positive
**118.064@5.030**	up	2.10	0.002	0.018	positive
**677.407@1.334**	up	1.94	0.002	0.018	positive
**835.103@1.346**	down	1.26	0.002	0.018	positive
**684.237@1.451**	up	2.80	0.002	0.018	positive
**609.848@13.474**	up	1.53	0.002	0.018	positive
**295.251@1.347**	down	1.22	0.003	0.023	positive
**311.245@1.402**	down	1.15	0.003	0.023	positive

**200.046@5.028**	up	2.27	0.003	0.023	positive
**250.219@1.350**	down	1.18	0.003	0.023	positive
**243.186@9.691**	up	1.16	0.003	0.023	positive
**312.274@8.872**	down	1.26	0.003	0.023	positive
**575.489@1.357**	up	1.22	0.003	0.023	positive
**889.205@1.346**	down	1.27	0.003	0.023	positive
**637.414@1.375**	up	3.05	0.003	0.023	positive
**82.044@1.285**	up	2.03	0.003	0.023	positive
**144.043@13.472**	up	1.39	0.003	0.023	positive
**335.222@1.371**	down	1.55	0.003	0.023	positive
**731.430@1.422**	up	1.87	0.003	0.023	positive
**665.662@1.360**	up	2.73	0.003	0.023	positive
**557.309@8.349**	up	2.01	0.003	0.023	positive
**627.415@1.363**	up	3.49	0.003	0.023	positive
**422.163@1.266**	up	3.31	0.001	0.026	negative
**424.161@1.266**	up	3.50	0.001	0.026	negative
**446.206@1.268**	up	4.12	0.001	0.026	negative
**450.158@1.415**	up	2.73	0.001	0.026	negative
**424.336@1.266**	up	4.79	0.001	0.026	negative
**449.181@1.261**	up	3.06	0.001	0.026	negative
**446.206@1.262**	up	4.46	0.001	0.026	negative
**422.783@1.268**	up	7.22	0.001	0.026	negative
**422.730@1.268**	up	5.75	0.001	0.026	negative
**642.183@1.464**	up	4.91	0.001	0.026	negative
**450.338@1.416**	up	3.42	0.001	0.026	negative
**286.119@4.311**	up	4.02	0.001	0.026	negative
**416.028@10.351**	up	3.24	0.001	0.026	negative
**374.073@10.325**	up	3.76	0.001	0.026	negative
**356.174@1.269**	up	4.59	0.001	0.026	negative
**424.780@1.260**	up	6.11	0.001	0.026	negative
**434.038@10.351**	up	3.60	0.001	0.026	negative
**850.822@1.268**	up	7.68	0.001	0.026	negative
**235.997@2.353**	up	4.04	0.001	0.026	negative
**426.167@1.265**	up	3.61	0.001	0.026	negative
**468.169@4.330**	up	1.90	0.001	0.026	negative
**450.410@1.416**	up	4.53	0.001	0.026	negative
**386.186@1.269**	up	4.89	0.001	0.026	negative
**432.198@1.265**	up	11.42	0.001	0.026	negative
**500.174@1.273**	up	4.45	0.001	0.026	negative
**246.009@7.121**	up	2.28	0.001	0.026	negative
**479.888@10.357**	up	3.39	0.001	0.026	negative

**898.728@1.261**	up	4.25	0.001	0.026	negative
**460.091@4.301**	up	14.40	0.001	0.026	negative
**386.041@4.301**	up	3.49	0.001	0.026	negative
**478.073@5.044**	up	6.00	0.001	0.026	negative
**510.233@1.225**	up	4.11	0.001	0.026	negative
**210.004@3.861**	down	3.46	0.001	0.026	negative
**476.215@2.809**	up	5.05	0.001	0.026	negative
**305.992@3.872**	down	2.58	0.001	0.026	negative
**780.203@1.449**	up	3.19	0.001	0.026	negative
**466.138@7.514**	up	4.02	0.001	0.026	negative
**498.171@1.246**	up	3.58	0.001	0.026	negative
**478.069@5.049**	up	4.88	0.001	0.026	negative
**530.090@5.032**	up	2.26	0.001	0.026	negative
**795.160@1.430**	up	3.44	0.001	0.026	negative
**374.010@5.049**	up	1.72	0.002	0.027	negative
**110.998@2.919**	down	1.53	0.002	0.027	negative
**556.106@7.113**	up	2.03	0.002	0.027	negative
**403.089@2.912**	down	1.56	0.002	0.027	negative
**191.917@7.124**	up	1.93	0.002	0.027	negative
**318.104@6.006**	up	2.95	0.002	0.027	negative
**445.102@1.444**	down	5.15	0.002	0.027	negative
**614.181@1.414**	up	2.28	0.002	0.027	negative
**610.197@6.008**	up	7.49	0.002	0.027	negative
**58.042@7.453**	up	1.31	0.002	0.027	negative
**596.221@1.432**	up	3.11	0.002	0.027	negative
**492.135@5.042**	up	1.93	0.002	0.027	negative
**614.182@1.414**	up	2.29	0.002	0.027	negative
**388.099@5.039**	up	1.78	0.002	0.027	negative
**382.075@7.279**	up	2.50	0.002	0.027	negative
**514.127@4.986**	up	2.70	0.002	0.027	negative
**451.990@5.046**	up	1.96	0.002	0.027	negative
**524.092@1.410**	up	1.92	0.002	0.027	negative
**184.050@4.293**	up	2.75	0.002	0.027	negative
**258.131@1.961**	up	1.95	0.002	0.027	negative
**200.044@5.046**	up	1.99	0.003	0.030	negative
**492.135@5.046**	up	1.95	0.003	0.030	negative
**742.247@1.458**	up	1.88	0.003	0.030	negative
**88.015@2.780**	up	2.43	0.003	0.030	negative
**191.916@5.047**	up	1.96	0.003	0.030	negative
**413.833@17.826**	up	1.05	0.003	0.030	negative
**334.046@10.373**	up	3.07	0.003	0.030	negative

**112.015@1.554**	up	2.45	0.003	0.030	negative
**200.168@5.048**	up	1.99	0.003	0.030	negative
**106.026@3.856**	down	2.55	0.003	0.030	negative
**386.068@2.750**	up	19.03	0.003	0.030	negative
**304.018@5.064**	up	1.79	0.003	0.030	negative
**594.088@4.925**	up	1.69	0.003	0.030	negative
**218.013@2.383**	up	2.12	0.003	0.030	negative
**431.105@5.042**	up	1.77	0.003	0.030	negative
**419.973@7.156**	up	1.77	0.003	0.030	negative
**629.473@1.341**	down	1.39	0.003	0.030	negative
**278.035@5.046**	up	2.70	0.003	0.030	negative
**315.277@1.439**	down	1.10	0.004	0.031	positive
**431.232@1.255**	down	1.72	0.004	0.031	positive
**414.204@1.258**	down	1.78	0.004	0.031	positive
**556.448@1.344**	down	1.18	0.004	0.031	positive
**574.604@9.700**	up	1.73	0.004	0.031	positive
**774.844@1.278**	up	2.39	0.004	0.031	positive
**265.227@1.385**	down	1.45	0.004	0.031	positive
**134.075@1.259**	down	1.42	0.004	0.031	positive
**307.198@9.729**	up	1.50	0.004	0.031	positive
**465.170@1.438**	up	1.31	0.004	0.031	positive
**216.110@10.420**	down	1.39	0.004	0.031	positive
**183.155@1.382**	down	1.59	0.004	0.031	positive
**462.476@1.131**	down	1.16	0.004	0.031	positive
**264.014@7.119**	up	1.95	0.004	0.039	negative
**514.093@1.270**	up	2.38	0.004	0.039	negative
**509.820@17.860**	up	1.05	0.004	0.039	negative
**333.238@1.351**	down	1.21	0.004	0.039	negative
**200.044@7.119**	up	1.87	0.004	0.039	negative
**341.995@7.123**	up	1.81	0.004	0.039	negative
**272.001@1.128**	down	1.07	0.004	0.039	negative
**500.121@5.060**	up	1.64	0.004	0.039	negative
**596.108@5.056**	up	1.95	0.004	0.039	negative
**376.008@5.046**	up	1.68	0.004	0.039	negative
**398.996@8.771**	down	1.17	0.004	0.039	negative
**582.407@1.339**	down	1.36	0.005	0.041	positive
**280.137@1.253**	down	1.77	0.005	0.041	positive
**488.113@1.435**	up	1.80	0.005	0.041	positive
**532.242@9.096**	up	1.07	0.005	0.041	positive
**375.256@9.354**	up	1.18	0.005	0.041	positive
**231.143@11.714**	up	1.26	0.005	0.041	positive

**117.040@8.488**	down	1.29	0.005	0.041	positive
**90.047@1.263**	down	2.69	0.005	0.041	positive
**150.135@1.369**	down	1.33	0.005	0.041	positive
**700.137@1.418**	up	1.67	0.005	0.041	positive
**176.031@2.785**	up	39.98	0.005	0.046	negative
**684.009@1.538**	up	62.14	0.005	0.046	negative
**404.196@1.422**	up	2.57	0.005	0.046	negative
**100.015@1.534**	up	1.80	0.005	0.046	negative
**210.037@1.998**	up	2.09	0.005	0.046	negative
**275.956@2.798**	up	21.38	0.005	0.046	negative
**419.152@5.090**	up	1.90	0.005	0.046	negative
**726.066@1.393**	up	6.12	0.005	0.046	negative
**257.946@2.783**	up	16.93	0.005	0.046	negative
**271.986@2.887**	down	1.59	0.005	0.046	negative
**364.982@2.818**	up	12.87	0.005	0.046	negative
**586.201@1.249**	up	2.15	0.005	0.046	negative
**726.063@1.393**	up	6.03	0.005	0.046	negative
**337.993@4.729**	down	1.50	0.005	0.046	negative
**530.198@1.419**	up	4.61	0.005	0.046	negative
**642.203@6.955**	up	1.91	0.005	0.046	negative
**482.181@1.290**	up	2.47	0.005	0.046	negative
**630.182@1.478**	up	3.10	0.005	0.046	negative
**618.325@4.233**	up	4.49	0.005	0.046	negative
